# Fertility History and Biomarkers Using Prospective Data: Evidence From the 1958 National Child Development Study

**DOI:** 10.1007/s13524-020-00855-x

**Published:** 2020-03-25

**Authors:** Maria Sironi, George B. Ploubidis, Emily M. Grundy

**Affiliations:** 1 Department of Social Science, University College London, 55-59 Gordon Square, London, WC1H 0NU United Kingdom; 2 UCL Center for Longitudinal Studies, University College London, 55-59 Gordon Square, London, WC1H 0NU United Kingdom; 3 Institute for Social & Economic Research, University of Essex, Wivenhoe Park, Colchester, Essex, CO4 3SQ United Kingdom; 4 Centre for Fertility and Health, Norwegian Institute for Public Health, Lovisenberggata 8, 0456 Oslo, Norway

**Keywords:** Biomarkers, Fertility, Parity, Age at childbearing, Health

## Abstract

Research on the later-life health implications of fertility history has predominantly considered associations with mortality or self-reported indicators of health. Most of this previous research has either not been able to account for selection factors related to both early-life and later-life health or has had to rely on retrospectively reported accounts of childhood circumstances. Using the 1958 National Child Development Study, and in particular the biomedical survey conducted in 2002–2003, we investigate associations between fertility histories (number of children and age at first and at last birth) and biomarkers for cardiometabolic risk and respiratory function in midlife among both men and women. Results from models that adjusted for a very wide range of childhood factors, including early-life socioeconomic position, cognitive ability, and mental health, showed weak associations between parity and biomarkers. However, we found an inverse association between age at first birth and biomarkers indicative of worse cardiometabolic health, with poorer outcomes for those with very young ages at entry to parenthood and increasingly better outcomes for those becoming parents at older ages. A very young age at last birth was also associated with less favorable biomarker levels, especially among women. Results highlight the value of prospectively collected data and the availability of biomarkers in studies of life course determinants of health in midlife and later.

**Electronic supplementary material:**

The online version of this article (10.1007/s13524-020-00855-x) contains supplementary material, which is available to authorized users.

## Introduction

Parenthood leads to major changes in activities, lifestyles, and allocation of resources; and pregnancy, parturition, and lactation involve considerable physiological changes for women. Long-established evolutionary theories of aging posit a negative relationship between fertility and longevity, reflecting trade-offs in investment in younger-age reproductive fitness and somatic maintenance in the post-reproductive period (Kirkwood and Rose 1991), and a growing literature has indicated linkages between fertility history and later-life health and mortality. In general, previous studies have indicated a J-shaped association between overall parity and mortality, with higher risks for childless and high-parity parents than for parents of two or three children (for reviews, see Högnäs et al. 2017; Hurt et al. 2006; Zeng et al. 2016). Although many studies have considered only women, those that have included men have tended to report similar albeit less strong associations suggesting underlying biosocial processes, as well as specific physiological effects that apply only to women. Timing of parenthood has been shown to be important, with many studies showing increased later-life mortality and poorer health outcomes among those entering parenthood at a young age, although with some contextual variations (Grundy and Foverskov 2016). Mechanisms underlying these associations are hypothesized to include a range of partly offsetting factors (Grundy and Tomassini 2005). On the positive side, children provide an incentive for healthier behaviors and a source of social interaction and support during both child-rearing and subsequent phases of life. Less positively, parenthood involves stresses, including the stress involved in pregnancy and childbearing for women, and substantial economic costs. For women, biological mechanisms include lowered estrogen exposure and harmful changes to lipid and glucose metabolism during pregnancy (Hardy et al. 2007). Additionally, studies have shown that women who give birth in their teens have higher risks of developing eclampsia, pregnancy-related hypertension, lasting insulin resistance, and altered cholesterol profiles (Lacey et al. 2017). Moreover, complications of pregnancy—including hypertension, gestational diabetes, preterm birth, and low birth weight—are associated with cardiometabolic risk (Hardy et al. 2007). In addition to these biological challenges for women, more general stresses related to child-rearing may also affect fathers. Cumulative effects of these stresses may outweigh salutogenic effects of parenthood, especially for young parents, who may be less resilient to stress and have fewer social and economic resources (Falci et al. 2010), and for those with closely spaced births (Grundy and Kravdal 2010) and large family sizes (D’Elio et al. 1997). Moreover, early parenthood may lead to disruption of educational and career progression and is associated with increased risk of partnership breakdown, all of which may increase risks of socioeconomic and social disadvantage (Grundy and Read 2015). Additionally, despite evidence that parenthood may be associated with less risky behaviors arising from social control exerted by partners and children and the motivation that parenthood brings to set a good example, some studies have suggested a positive association with obesity (Sowers 2003).

An important complicating factor is the need to account for selection to particular fertility pathways. Epigenetic and hormonal influences prompted by unstable environments in childhood may lead to earlier sexual maturation, sexual debut, and poorer choice of partners (Waynforth 2012), and numerous studies have shown that early parenthood is associated with childhood disadvantage (Kiernan 1997; Sigle-Rushton 2005) as well as with disadvantageous health behaviors and health outcomes across the life course (Henretta 2007; Nyström Peck 1994). The complexity of these associations means that our understanding of underlying processes and mechanisms is still limited.

The aim of this study is to contribute to our understanding of these processes by examining associations between aspects of fertility histories and midlife biomarkers indicative of health status, which may mediate progression to later disability and mortality. On the one hand, positive effects of parenthood (such as the social control of health-related behaviors) may imply, for example, less smoking among parents. Parents, and parents with more children, may also receive greater social support than childless or low-parity individuals (Grundy and Read 2015), which may buffer health-damaging effects of stress. These mechanisms imply lower cardiometabolic risks and better respiratory function among parents than among childless individuals as well as possibly increased benefits of having a larger family size. On the other hand, the negative effects of some patterns of childbearing and child-rearing (such as greater stress associated with early parenthood and high parity) may have negative implications for these indictors of health. For example, as noted earlier, higher parity has been found to be associated with higher risks of obesity, a well-established cardiometabolic risk factor (Smith 2003; Sowers 2003).

One of the main problems besetting efforts at unravelling these associations is that early-life socioeconomic and health factors are known to be strongly associated both with fertility trajectories and with later-life health. Many previous studies have either not been able to take account of these selection processes or had to rely on rather limited accounts of childhood circumstances reported retrospectively by older adults (Grundy and Foverskov 2016; Grundy and Read 2015). We use high-quality prospective data from the 1958 National Child Development Study (NCDS), combining information from Sweeps 0 (1958) to 7 (2004; age 46) and from a biomedical survey collected in 2002 (age 44). We examine aspects of parenthood trajectories, including parity and age at first and at last birth, and their associations with midlife biomarkers indicative of cardiometabolic risk and respiratory function. Our aim is to investigate the extent to which associations between fertility history indicators and these related outcomes show a consistent pattern, suggestive of a common underlying mechanism of action.

## Previous Research

The literature on associations between fertility and later-life health has focused partly on the association between the number of children and all-cause or cause-specific mortality (Dior et al. 2013; Doblhammer 2000; Grundy and Kravdal 2007, 2010; Grundy and Tomassini 2006; Hinkula et al. 2005; Hurt et al. 2006; Jaffe et al. 2009, 2011; Tamakoshi et al. 2010) and has generally shown a J-shaped relationship between parity and mortality. Individuals who have two or three children have a lower mortality risk than those who are childless, have one child, or have four to five or more children. Many studies are restricted to women, but results from those also including men suggest that although associations are weaker, the J-shaped association is still observed. The similarity of findings for men and women suggests underlying biosocial pathways (Grundy and Kravdal 2007). Cause-specific mortality analyses provide some insights into possible underlying mechanisms. For example, results from Norway and Sweden showed inverse associations between parity and deaths from lung cancer, alcohol-related causes, and accidents and violence, with the highest mortality rates among childless individuals (Barclay et al. 2016; Grundy and Kravdal 2010). Mortality from these causes is strongly related to smoking, heavy alcohol use, and other risky behaviors, which would suggest that a lack of social control (from children) of health behaviors is one possible underlying mechanism. However, selection may be as or more important given that those who have experienced early disadvantage and/or developed poor health behaviors in adolescence may include groups less likely to find a partner and form a family.

Early disadvantage is also associated with a higher risk of early parenthood, as shown in other studies investigating the association later-life health and fertility history (Grundy and Kravdal 2014) or combined effects of fertility and partnership histories (Kravdal et al. 2012). Many studies that investigated associations between timing of fertility and later-life health indicated that early parenthood is associated with a higher risk of later-life mortality. This relationship is partially explained by socioeconomic background, health-related factors, and (in some settings) ethnicity (Grundy 2009; Kravdal et al. 2012; Spence and Eberstein 2009). However, analyses controlling for these influences, including sibling comparison studies (Barclay et al. 2016; Einiö et al. 2015), have also found an adverse association between early parenthood and later mortality risks, especially risks associated with poor health behaviors (e.g., lung cancer, accidents and violence), as for the childless (Barclay et al. 2016; Grundy and Kravdal 2010).

Other research has focused on associations between fertility and health outcomes, rather than mortality, both in midlife and at older ages (Buber and Engelhardt 2008; Grundy and Holt 2000; Grundy and Tomassini 2005; Gunes 2016; Hank 2010; Hanson et al. 2015; Henretta 2007; O’Flaherty et al. 2016; Pirkle et al. 2014; Read et al. 2011; Williams et al. 2011). Outcomes investigated include self-reported health, disability, presence of limiting long-term illness, chronic diseases, allostatic load, grip strength, psychological well-being, and mental health (Grundy and Read 2015; Henretta et al. 2008; Keenan and Grundy 2018; Spence 2008). Findings are similar to those reported for mortality, with early parenthood, childlessness, and high parity associated with poorer health outcomes. Also, some studies have indicated that later age at first parenthood is associated with better health later in life (Grundy and Tomassini 2005; Read and Grundy 2011). As for mortality, studies have indicated that the relationship between fertility and health is partially confounded or mediated by life course socioeconomic factors and partnership status, with some evidence of contextual influences (Grundy and Foverskov 2016; Grundy and Read 2015).

Most of these studies of associations between fertility histories and health have relied on self-reported indicators. These measures have some limitations: for example, they may be influenced by health expectations, which are also correlated with socioeconomic status (Daltroy et al. 1999; Jürges 2007; Quesnel-Vallee 2007). Recently, other measures have become available thanks to the collection of blood samples and observer-measured indicators collected in nurse-administered survey modules. However, relatively few studies to date have looked specifically at the association between fertility history and biomarkers. Lawlor et al. (2003), in analyses of data for men and women aged 60–79 included in the British Regional Heart Study and the British Women’s Health and Heart Study, examined associations between parity and a range of coronary heart disease (CHD) risk factors, including systolic and diastolic blood pressure, obesity, lipid profiles, and diabetes. Results showed an association between increasing number of children and increasing obesity among both women and men, although this was attenuated among men after the researchers controlled for adult lifestyle and socioeconomic indicators. Among women, higher parity was also associated with metabolic risk factors. These investigators concluded that lifestyle factors associated with having large families may be associated with obesity and CHD risk in both women and men and that women may suffer additional influences arising from biological changes associated with pregnancy, such as lipid and glucose metabolism and adverse coagulation. Hardy et al. (2007) used data from the 1946 British birth cohort to investigate the association between number of children and CHD risk factors, using blood pressure, body mass index (BMI), waist-to-hip ratio (WHR), total cholesterol, high-density lipoprotein and low-density lipoprotein cholesterol and triglyceride levels, and glycated hemoglobin at age 53. They found that BMI, WHR, and type 2 diabetes in women, and glycated hemoglobin in men showed a linearly increasing trend with increasing number of children; however, the authors found no associations with other outcomes investigated and concluded that the associations observed were mostly explained by behavior and lifestyle. Grundy and Read (2015) looked at the link between retrospectively collected fertility histories and allostatic load (and long-term illness) among people born before 1952 in England. The measure of allostatic load was derived using nine biomarkers: systolic and diastolic blood pressure, WHR, peak expiratory flow, HDL/total cholesterol ratio (mg/dL), triglycerides, glycated hemoglobin, fibrinogen, and C-reactive protein. They found that earlier ages at first birth were associated with worse allostatic load, with the relationship mediated in part by wealth, physical activity, and smoking. No association between childlessness and allostatic load was found. Lacey et al. (2017) used data from the 1958 NCDS to look specifically at the association between age at first birth and biomarkers representing cardiovascular risk factors and found that experiencing a teenage birth was associated with an adverse cardiovascular profile in midlife.

Thus, results of research focusing on biomarkers and observer-measured health indictors are not always consistent with findings from studies using self-reported health outcomes; generally, studies using self-reported measures have reported a stronger association between parity and health than studies using biomarkers. We build on this past research and analyze associations between fertility histories and several biomarkers of cardiometabolic and respiratory function. These markers are related to health behaviors and experiences of cumulated stress (Dariotis et al. 2011; Umberson et al. 2010) and thus are plausibly linked to social control of behaviors and provision of social support mechanisms hypothesized to underlie linkages between fertility history and health. We thus aim to contribute to elucidating the mechanisms underlying associations between fertility histories and later health.

## Data and Methods

### Data

Data used for the analysis are taken from waves of the 1958 NCDS. The NCDS started in 1958 with more than 17,000 infants born in England, Scotland, and Wales in a single week in March 1958. Ten additional sweeps have been collected since 1958 when respondents were aged 7, 11, 16, 23, 33, 42, 46, 50, and 55. The study has collected information on multiple domains, such as physical and educational development, economic circumstances, employment, family life, and health behavior. In 2002, when respondents were 44–45 years old, biomedical data were collected for more than 9,000 respondents. Blood samples were collected from 88% of those examined, and 8,018 blood samples were received from subjects who gave consent to extraction of DNA. In this study, we used biomarkers obtained from this survey and combine these data with information collected in other sweeps on fertility histories (at ages 23, 33, 42, and 46) and information collected in childhood on early-life health, socioeconomic conditions, and cognitive ability.

A complete case sample including respondents who provided blood samples, information on fertility histories, and all background variables comprised only 2,289 individuals (51.2% women). Analyses using this selected sample would be subject to various selection biases reflecting differential loss to follow-up. To mitigate this, we undertook multiple imputation analysis, allowing us to retain a sample of 15,252 respondents for the parity analysis (49.2% women) and 11,754 for age at first and last birth (51.5% women). Further information on multiple imputation and results using the complete case sample are reported in the online appendix.

### Methods

#### *Measures of Health*

To study the relationship between fertility and health, we selected specific health indicators related to potential mechanisms underlying the associations between fertility histories and later health identified in the previous literature. In particular, we selected measures related to accumulated stress and/or health-related behavior (e.g., smoking and eating habits). Hence, the analysis focuses on markers of two major health outcomes: cardiovascular and respiratory function.

We used the following biomarkers used to investigate cardiometabolic risk:

- *Fibrinogen*, a protein that helps coagulation, is a marker of inflammation and cardiovascular disease (g/L). A normal range of fibrinogen is between 2.0 and 4.0 g/L. Very low and high levels of fibrinogen are associated with the presence of several disorders (e.g., blood clotting, various forms of cancer).
- *C-reactive protein* (CRP) is also a protein that serves as an indicator of inflammation and cardiovascular disease (g/L).^1^ A normal range of CRP is between 1.0 and 3.0 g/L. High levels of CRP indicate inflammation, infection, trauma, necrosis, malignancy, and allergic reaction.
- *Glycated hemoglobin (HbA1c)* is an index of glucose metabolism over the previous 30−90 days, and high levels (>6%) are indicative of the presence of diabetes mellitus.
- *Cholesterol ratio* is the ratio between total cholesterol and HDL; high values (>3.5) of this ratio indicate a higher risk for cardiovascular disease.
- *High blood pressure* was assessed with three measures of systolic and diastolic blood pressure (BP). The mean of valid readings was used, and an individual was recorded as having high blood pressure if the average value was above 140/90 mmHg; high blood pressure is a risk factor for circulatory diseases.
- *Obesity* is defined as body mass index (BMI), calculated using information on measured height and weight, greater than 30; obesity is also associated with higher cardiovascular disease risk.
- *Waist-to-hip ratio* (WHR) is the ratio of waist to hip circumference. A normal WHR should be less than 0.9 for men and 0.85 for women, according to World Health Organization (WHO) guidelines (World Health Organization 2011).
- *Metabolic syndrome* is defined by the new International Diabetes Federation as having central obesity (defined as waist circumference ≥94cm for men and ≥80cm for women), plus any two of the following four factors: raised triglyceride levels ≥150 mg/dL (1.7 mmol/L), reduced HDL cholesterol <40 mg/dL (1.03 mmol/L) in males and <50 mg/dL (1.29 mmol/L) in females, raised blood pressure (systolic BP ≥130 or diastolic BP ≥85 mmHg), and raised fasting plasma glucose ≥100 mg/dL (5.6 mmol/L).

As indicators of respiratory function, we used the ratio between forced expiratory volume (FEV1, highest measurement used as a valid one) and forced vital capacity (FVC). FEV1 is a measure of how much air a person can exhale during forced breath during the first second. FVC is the volume of air that can forcibly be blown out after full inspiration. FEV1/FVC is used in the diagnosis of obstructive and restrictive lung disease—FEV1/FVC <0.7 indicates chronic obstructive lung disease—and is the standard biomarker indicative of smoking-related damage to lung function.

#### *Fertility History*

To have a broad picture of individuals’ fertility trajectories, we considered not only the number of natural children (by age 44) but also age at first birth and age at last birth. This was the last birth before the biomedical survey conducted when respondents were aged 44, so it is possible that some individuals (particularly men) in the sample had children subsequently.^2^ Given that previous studies have indicated a nonlinear relationship between parity and health, we used a categorical variable for number of children with five possible values: childless, one child, two children, three children, and four or more children. Age at first birth was categorized into bands that are slightly different for men and women, based on results from previous studies (Grundy and Read 2015; Hobcraft 2008; Read et al. 2011) and confirmed by the distribution in our sample: before age 20, 20–24, 25–29, 30–34, and after age 35 for women; before age 23, 23–27, 28–32, 33–38, and after age 39 for men. Age at last birth is also a categorical variable with five possible values (the same for men and women): before age 25, 25–29, 30–34, 35–39, and after age 40.

#### *Control Variables*

Health outcomes and fertility variables may be influenced by confounders that need to be considered in the analysis of associations between health and fertility. In particular, as noted earlier, childhood health and socioeconomic circumstances have been found to be associated with both adult health (Goodman et al. 2011; Haas 2007, 2008; Nyström Peck 1994; Rahkonen et al. 1997) and fertility trajectories, especially timing of birth (Geronimus and Korenman 1992; Penman-Aguilar et al. 2013). Specific exposures in childhood are associated with cardiometabolic risk and respiratory function in midlife, making it even more important to control for these influences. For example, living in damp or overcrowded housing as a child increases risks of poor respiratory function later in life (Bartley et al. 2012). Using information collected at age 0, 7, 11, and 16, we were able to take account of early-life health and socioeconomic background.^3^ We considered social class at birth, based on father’s occupation: manual (skilled manual, partly skilled, unskilled, and other/unknown) versus nonmanual (i.e., professional, managerial, and skilled nonmanual), parental years of education (either mother’s or father’s, whichever was higher if both were available), and whether the respondent’s mother had stayed in school beyond the minimum leaving age. We also considered living conditions at age 11: financial hardship during the past year, measured as overcrowding in the household (more than 1.5 persons per room) and lack of access to housing amenities (lacking access to a bathroom, and/or an indoor toilet, and/or cooking facilities, and/or hot water, vs. having access to all). These measures were all based on reports from respondents’ parents. In order to take into account respondents’ health in childhood, we included birth weight; whether the mother smoked during pregnancy; whether the child was out of school for a month or more because of health problems; number of hospital admissions before age 11; episodes of enuresis at ages 7 and 11; and poor physical coordination at age 11. We also included mental health at ages 7 and 11 as indicated by the Bristol Social Adjustment Guide score completed by the teacher^4^ at age 11 and the Rutter Behaviour Scales completed by the mother at ages 7 and 11.^5^ We also considered possible family disruptions during childhood by including a variable indicating family difficulties (i.e., divorce, separation, and desertion) at age 7 and another variable indicating parental divorce before age 11. To take risky health behaviors during adolescence into account, we considered whether the respondent was smoking at age 16. Moreover, we included a measure of cognitive ability at age 11 (i.e. a general ability test score^6^ consisting of 40 verbal and 40 nonverbal items), an indicator of need for special education at age 11, and parents’ interest in the respondent’s education at age 11 (interested vs. not interested). Finally, we included cohort members’ ethnicity, level of education at age 23, number of partnerships (marriages and cohabitations) by age 42 (none, one, or more than one), whether ever unemployed between January 1978 and December 2001, and age at the biomedical interview.^7^

#### *Analytic Strategy*

We report findings stratified by gender given that pregnancy and childbearing are specific to women, and some physiological mechanisms possibly underlying associations between fertility and later health accordingly also apply only to women (Lawlor et al. 2003). In addition, women’s generally greater child-rearing responsibilities also suggest that linkages between fertility patterns and health might vary by gender.

We first produce descriptive statistics for the variables used in the analysis. To maximize representativeness, the descriptive statistics are based on the specific sample available at the sweep in which the variable was collected (e.g., the sample available at the biomedical sweep for biomarkers). For variables built using multiple sweeps, we also consider the sample for which the information is available at the time of the biomedical sweep. Before estimating multivariable regression models, we performed multiple imputation with chained equations with 80 imputed data sets, using all variables in the substantive model as well as auxiliary variables (see the online appendix) in the imputation process (Carpenter and Kenward 2012). Multiple imputation operates under the missing at random (MAR) assumption (Little and Rubin 2014), which in this case implies that our estimates are valid if missingness is due to variables included in the imputation phase.

We then fitted multivariable linear or logistic (depending on the outcome) regression models for all our health measures and each of the fertility characteristics only for cases with nonmissing outcomes, controlling for all the aforementioned variables as suggested in the literature (von Hippel 2007). An analysis retaining the imputed values of the outcomes returned similar results (results available from the corresponding author).

Given the richness and the prospective nature of the NCDS, we included two additional sets of analysis on all outcomes and exposures (available in the online appendix): a regression analysis without any confounders, and a regression analysis including a limited set of confounders similar to those included in previous studies. In this intermediate model, we included the following confounders: father’s social class at birth, parents divorced at age 11, overcrowding at age 11, housing conditions at age 11, teenage smoking, number of times hospitalized by age 11, out of school for more than a month at age 11, parents’ years of education, education level, number of partnerships, and ever unemployed between 1978 and 2002. Results from this intermediate model can be more easily compared with the previous literature. The full models—presented in the main text—include additional confounders that are unique to the NCDS and to prospective data, such as mental health and behavioral measures at ages 7 and 11, and cognitive ability at age 11 (Jivraj et al. 2017).

Finally, to detect potential unmeasured confounding and measurement error, we used the negative controls technique designed to detect both suspected and unsuspected sources of spurious causal inference (Lipsitch et al. 2010). Negative controls test the ability of the controls included in the regressions to produce a null association between the exposure and the negative control outcome, taking into account that there is no plausible mechanism of action that links them other than confounding and/or measurement error. As negative controls, we used variables from the 2002/2003 biomedical survey: *hair color* (light brown and blond vs. dark brown and black), *ear tested first* (left vs. right), and *arm blood taken from* (left vs. right). There is no known mechanism of action that could potentially link fertility behavior with these negative control outcomes; however, unknown mechanisms (such as genetic predisposition related to both fertility behavior and hair color) might still produce spurious associations. Furthermore, the use of negative controls serves as sensitivity analysis for measurement error. It is well known that nondifferential, random, or systematic measurement error in the exposure can bias parameter estimates, which is also the case for differential error in the outcome (Armstrong 1998). Furthermore, measurement error in the confounders included may give rise to residual confounding bias. We believe that in our study, residual confounding is more likely because fertility behavior is not likely to suffer from severe measurement error, nor it is likely to influence error in the measurement of biomarkers. Therefore, any association between fertility behavior and the three negative control outcomes would most likely reflect unknown unmeasured confounders or residual confounding due to measurement error. The results are reported in the online appendix.

## Results

### Descriptive Statistics

Table 1 reports information on health indicators of cardiometabolic risk and respiratory function. The average level of fibrinogen in our sample is in the normal range and is slightly higher for women than for men. Average values of CRP and of glycated hemoglobin were also in the normal range.

**Table 1:** Health measures: Univariate descriptive statistics

	Men			Women		
	Mean or %	SD	*N*	Mean or %	SD	*N*
Cardiometabolic Risk Factors						
Fibrinogen (g/L)	2.88	0.58	3,845	3.03	0.65	3,838
CRP (g/L)	1.97	4.40	3,856	2.38	4.16	3,836
Glycated hemoglobin	5.32	0.76	3,976	5.19	0.63	3,947
Total cholesterol	6.07	1.14	3,927	5.70	1.00	3,897
HDL cholesterol	1.43	0.34	3,914	1.69	0.41	3,894
LDL cholesterol	3.57	0.93	3,571	3.29	0.87	3,820
Cholesterol ratio (total/HDL)	4.42	1.19	3,914	3.54	1.00	3,893
% With high blood pressure	16.0		4,608	5.58		4,622
BMI	27.8	4.27	4,585	26.9	5.53	4,625
% Obese	25.3		4,585	23.5		4,625
WHR	0.93	0.06	4,629	0.81	0.06	4,670
% Obese using WHR	34.0		4,629	25.1		4,670
% With metabolic syndrome	45.1		4,143	26.1		4,149
Respiratory Functions						
FEV1/FVC	0.78	0.12	4,522	0.78	0.12	4,568
*N* in Biomedical Survey	4,665			4,712		

On average, men’s cholesterol ratio was higher than normal (~3.5); 16% of men had high blood pressure, compared with only 5.6% of women. Between 23% and 25% of the sample was obese according to their BMI, but 25% of women and 34% of men were obese according to the WHR. More men than women had measures indicative of metabolic syndrome: 45.2% and 26.1%, respectively. The ratio between forced expiratory volume and forced vital capacity was 78% for both men and women.

In Table 2, we report the correlation matrix for all the biomarkers included in the analysis, separately for men and women. The highest correlation is between fibrinogen and CRP for both men and women (.591 and .547, respectively). As would be expected, there was also a substantial correlation between obesity as indicated by BMI and WHR, cholesterol ratio, and metabolic syndrome, especially among men. The correlations among the other variables were weaker—on average, less than .3. This shows the importance of examining different biomarkers separately instead of considering only a combination of these as a general indicator of health.

**Table 2:** Correlation among biomarkers

	ln(Fibrinogen)		ln(CRP)		Glycated Hemoglobin			Cholesterol Ratio	High Blood Pressure	Obese	WHR	Metabolic Syndrome	FEV1/FVC
Men													
ln(fibrinogen)	1.000												
N	3,845												
ln(CRP)	**.591**		1.000										
N	3,845		3,856										
Glycated hemoglobin	**.158**		**.176**		1.000								
N	3,806		3,817		3,976								
Cholesterol ratio	**.180**		**.234**		**.143**			1.000					
N	3,765		3,771		3,864			3,914					
High blood pressure	**.041**		**.091**		**.045**			.024	1.000				
N	3,818		3,829		3,948			3,887	4,608				
Obese	**.124**		**.269**		**.151**			**.256**	**.119**	1.000			
N	3,798		3,809		3,928			3,867	4,552	4,585			
WHR	**.209**		**.356**		**.213**			**.326**	**.133**	**.474**	1.000		
N	3,828		3,839		3,959			3,896	4,593	4,579	4,629		
Metabolic syndrome	**.131**		**.259**		**.211**			**.363**	**.189**	**.415**	**.540**	1.000	
N	3,781		3,791		3,913			3,871	4,127	4,102	4,137	4,143	
FEV1/FVC	–.030		–.018		–.012			.014	.022	.027	.009	–.001	1.000
N	3,755		3,766		3,877			3,821	4,487	4,469	4,509	4,041	4,522
Women													
ln(fibrinogen)	1.000												
N	3,838												
ln(CRP)	**.547**		1.000										
N	3,836		3,836										
Glycated hemoglobin	**.225**		**.209**		1.000								
N	3,801		3,799		3,947								
Cholesterol ratio	**.352**		**.349**		**.258**			1.000					
N	3,756		3,754		3,847			3,893					
High blood pressure	.027		**.073**		.024			**.042**	1.000				
N	3,781		3,779		3,887			3,836	4,622				
Obese	**.298**		**.430**		**.228**			**.335**	**.070**	1.000			
N	3,782		3,780		3,888			3,836	4,561	4,625			
WHR	**.242**		**.366**		**.225**			**.406**	**.094**	**.360**	1.000		
N	3,818		3,816		3,927			3,874	4,601	4,618	4,670		
Metabolic syndrome	**.264**		**.373**		**.311**			**.527**	**.156**	**.429**	**.483**	1.000	
N	3,761		3,759		3,868			3,850	4,108	4,101	4,144	4,149	
FEV1/FVC	.004		–.006		.003			.010	–.012	**.042**	–.011	.007	1.000
N	3,740		3,738		3,844			3,790	4,497	4,501	4,543	4,040	4,568

*Note:* Bold values denote correlations that are significant at 5% level.

Table 3 describes the fertility histories available for men and women at the time of the biomedical sweep. Most men and women had a child: 80.6% of men and 85.2% of women. On average, they had 1.73 and 1.90 children, respectively, with 36.4% of men and 40.8% of women having two children. Approximately 3% of both men and women had twins.

**Table 3:** Fertility measures: Univariate descriptive statistics

	Men		Women	
	Mean or %	SD	Mean or %	SD
% Ever Had a Child	80.6		85.2	
Number of Children	1.73	1.24	1.90	1.22
0	19.7		14.9	
1	20.6		18.2	
2	36.4		40.8	
3	16.3		18.1	
4+	7.0		8.0	
% With Twins	2.48		2.75	
Age at First Birth (mean)	27.9	5.70	25.4	5.49
Age at First Birth: Distribution (%)				
<23 (M) / <20 (W)	22.4		16.8	
23–27 (M) / 20–24 (W)	31.4		35.6	
28–32 (M) / 25–29 (W)	26.5		27.3	
33–38 (M) / 30–34 (W)	15.6		14.1	
39+ (M) / 35+ (W)	4.1		6.1	
Age at Last Birth (mean)	32.1	5.34	30.1	5.32
Age at Last Birth: Distribution (%)				
<25	9.2		17.7	
25–29	27.2		32.4	
30–34	33.7		30.4	
35–39	21.3		15.8	
40+	8.7		3.8	
*N* With Information on Fertility History at Biomedical Sweep	6,048		6,356	
Age at first birth	4,854		5,407	
Age at last birth	3,608		4,253	

Age at first birth was 2.5 years higher for men (27.9) than for women (25.4). One-third of men had their first child at ages 23–27, and one-third of women had their first child at ages 20–24. Age at last birth was two years higher for men (32.1 for men and 30.1 for women), and 33.7% of men and 30.4% of women had their last child when they were aged 30–34.

Descriptive statistics for the confounders included in the analysis are reported in the online appendix.

### Fertility and Cardiometabolic Risk Factors and Respiratory Volume

In this section, we present the results of multivariable regressions for cardiometabolic risk and respiratory function. All tables and graphs in this section report the coefficients for each fertility characteristic separately given that the regressions were run separately, and all the regression models include the full set of control variables.^8^

#### *Number of Children*

**Fig. 1:**
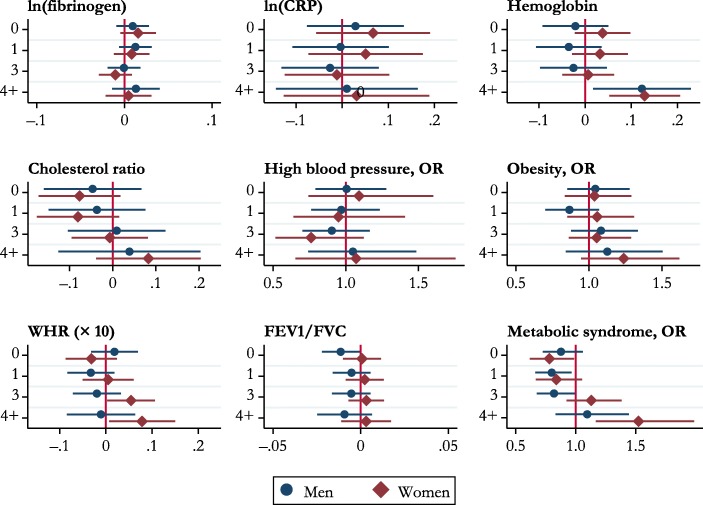
Number of children (ref. = two children) and biomarkers. Horizontal lines represent 95% confidence intervals.

Table 4 and Fig. 1 report results from the analysis of associations between parity and biomarkers. As shown in the table, these associations are very weak. Men and women with four or more children had a higher level of glycated hemoglobin (*B* = 0.123, *p* < .05, and *B* = 0.139, *p* < .05, respectively) than parents of two children. WHR was also higher for mothers of three to four or more children (i.e., *B* = 0.054, *p* < .05 and *B* = 0.078, *p* < .05, respectively) than for mothers of two children. Childless women had a lower probability of metabolic syndrome (OR = 0.78), and mothers of four or more children had a higher probability (OR = 1.524, *p* < .05). Among men, those who had one or three children had a lower risk of having metabolic syndrome (OR = 0.800, *p* < .05 and OR = 0.819, *p* < .05, respectively) than fathers of two. However, the results of the negative control analysis (see the online appendix, Figs. A7–A9) show that the observed associations between number of children, glycated hemoglobin, and metabolic syndrome (for women) may be due to bias arising from unmeasured confounders and/or measurement error: associations between hair color and having more than four children were observed.

**Table 4:** Multivariate regression models: Biomarkers and number of children

	log(Fibrinogen)		log(CRP)		Glycated Hemoglobin		Cholesterol Ratio (Total/HDL)	
	Men B/CI	Women B/CI	Men B/CI	Women B/CI	Men B/CI	Women B/CI	Men B/CI	Women B/CI
Number of Children (ref. = 2 children)								
0	0.009	0.016	0.029	0.067	–0.021	0.038	–0.046	–0.077
	–0.01,0.03	–0.00,0.04	–0.08,0.13	–0.06,0.19	–0.09,0.05	–0.02,0.10	–0.16,0.07	–0.17,0.02
1	0.012	0.008	–0.003	0.051	–0.035	0.032	–0.036	–0.080^†^
	–0.01,0.03	–0.01,0.03	–0.11,0.10	–0.07,0.17	–0.11,0.04	–0.03,0.09	–0.15,0.08	–0.18,0.02
3	–0.001	–0.01	–0.026	–0.011	–0.025	0.007	0.009	–0.007
	–0.02,0.02	–0.03,0.01	–0.13,0.08	–0.12,0.10	–0.10,0.05	–0.05,0.06	–0.10,0.12	–0.09,0.08
4+	0.013	0.005	0.01	0.031	0.123*	0.129*	0.039	0.083
	–0.01,0.04	–0.02,0.03	–0.14,0.16	–0.13,0.19	0.02,0.23	0.05,0.21	–0.13,0.20	–0.04,0.20
*N*	3,699	3,713	3,709	3,712	3,824	3,820	3,770	3,766

	High Blood Pressure		Obesity		WHR **×** 10		FEV1/FVC		Metabolic Syndrome	
	Men OR/CI	Women OR/CI	Men OR/CI	Women OR/CI	Men B/CI	Women B/CI	Men B/CI	Women B/CI	Men OR/CI	Women OR/CI
Number of Children (ref. = 2 children)										
0	1.006	1.092	1.044	1.037	0.019	–0.031	–0.011^†^	0.001	0.877	0.780*
	0.79,1.28	0.74,1.60	0.85,1.28	0.83,1.29	–0.03,0.07	–0.09,0.02	–0.02,–0.00	–0.01,0.01	0.73,1.06	0.62,0.99
1	0.97	0.95	0.866	1.056	–0.033	0.005	–0.005	0.002	0.800*	0.839
	0.76,1.23	0.64,1.41	0.70,1.07	0.85,1.31	–0.08,0.02	–0.05,0.06	–0.02,0.01	–0.01,0.01	0.66,0.97	0.67,1.05
3	0.904	0.762	1.082	1.053	–0.019	0.054*	–0.005	0.003	0.819*	1.13
	0.70,1.16	0.52,1.12	0.88,1.34	0.86,1.29	–0.07,0.03	0.00,0.11	–0.02,0.01	–0.01,0.01	0.68,0.99	0.92,1.38
4+	1.049	1.072	1.125	1.237	–0.011	0.078*	–0.009	0.003	1.096	1.524*
	0.74,1.49	0.65,1.76	0.84,1.50	0.95,1.62	–0.08,0.06	0.01,0.15	–0.02,0.01	–0.01,0.02	0.83,1.44	1.17,1.99
*N*	4,424	4,470	4,405	4,473	4,444	4,517	4,343	4,421	3,980	4,011

*Notes:* Included confounders are social class at birth, parental years of education, mother stayed at school beyond the minimum leaving age, financial hardship at age 11, overcrowding at age 11, housing conditions at age 11, birth weight, mother smoked during pregnancy, child was out of school for a month or more because of health problems, number of hospitalizations before age 11, enuresis at ages 7and 11, poor physical coordination at age 11, mental health at ages 7 and 11, Rutter Behaviour Scales completed by the mother at child’s ages 7 and 11, family difficulties at age 7, parental divorce before age 11, smoking at age 16, cognitive ability at age 11, need for special education at age 11, parents’ interest in the respondent’s education at age 11, ethnicity, level of education at age 23, number of partnerships by age 42, ever unemployed between January 1978 and December 2001, and age at the biomedical interview.

^†^*p* < .10; *p < .05

No other biomarkers showed an association with parity once all the control variables were included in the models. The associations between parity and most of the biomarkers observed in initial unadjusted models, and in models with a restricted number of confounders, (Fig. A1 and Fig. A2 in the online appendix) largely disappeared when the variables indictive of mental health and cognitive abilities in childhood were added to the model, in addition to the indicators of early-life socioeconomic background, childhood health, and adult socioeconomic characteristics.

#### *Age at First Birth*

**Fig. 2:**
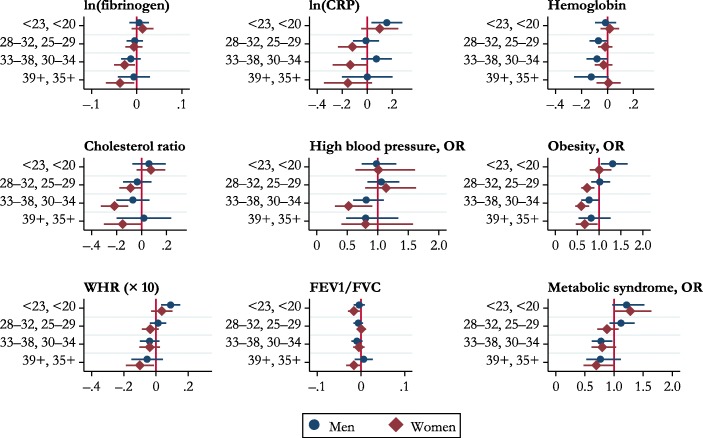
Age at first birth (ref. = 23–27 for men, and 20–24 for women) and biomarkers. Along the *y*-axis, ages for men are listed first, followed by ages for women. Horizontal lines represent 95% confidence intervals.

Table 5 and Fig. 2 show the association between age at first parenthood and biomarkers for cardiometabolic risk and respiratory function (taking into account all the aforementioned confounders). Women having their first child after age 30 had a lower level of fibrinogen. Levels of CRP were 15.7% (*p* < .05) higher for men having their first child before age 23. For women, CRP decreased monotonically with older age at first birth. Glycated hemoglobin was lower for men who had their first child after age 28. Having the first child at age 30–34 or 35 and older was associated with a lower cholesterol ratio (*B* = –0.217, *p* < .05 and *B* = –0.152, *p* < .05, respectively) and lower risk of high blood pressure^9^ (30–34: OR = 0.52, CI = [0.30, 0.91]) among women. Age at first birth was also associated with obesity. Among women, having the first baby after age 25 was associated with a lower probability of being obese than earlier first motherhood. Among men, having the first child before age 23 was associated with a higher risk of obesity, and becoming a father at ages 33–38 was associated with a lower risk of obesity. WHR was slightly higher for men having their first child before age 23 (*B* = 0.091, *p* < .05) and lower for women having their first child after age 35 (*B* = –0.1, *p* < .05). Men who had their first child at ages 33–38 and women who had their first child after age 30 had a lower risk of metabolic syndrome. Expiratory volume was worse for women who had had a teenage birth (*B* = –0.016, *p* < .05).

**Table 5:** Multivariate regression models: Biomarkers and age at first birth

	log(Fibrinogen)		log(CRP)		Glycated Hemoglobin		Cholesterol Ratio (Total/HDL)	
	Men B/CI	Women B/CI	Men B/CI	Women B/CI	Men B/CI	Women B/CI	Men B/CI	Women B/CI
Age at First Birth (ref. = 23–27 (M) / 20–24 (W))								
<23 (M) / <20 (W)	0.005	0.013	0.157*	0.100	–0.015	0.018	0.059	0.075
	–0.02,0.03	–0.01,0.04	0.03,0.28	–0.05,0.25	–0.10,0.07	–0.05,0.09	–0.07,0.19	–0.04,0.19
28–32 (M) / 25–29 (W)	–0.004	–0.006	–0.01	–0.118*	–0.069*	–0.017	–0.034	–0.088^†^
	–0.02,0.01	–0.03,0.01	–0.11,0.09	–0.23,–0.00	–0.14,–0.00	–0.07,0.04	–0.15,0.08	–0.18,0.00
33–38 (M) / 30–34 (W)	–0.013	–0.027*	0.074	–0.135^†^	–0.081*	–0.03	–0.069	–0.217*
	–0.03,0.01	–0.05,–0.00	–0.05,0.20	–0.28,0.01	–0.16,–0.00	–0.10,0.04	–0.20,0.06	–0.33,–0.11
39+ (M) / 35+ (W)	–0.007	–0.037*	0.001	–0.155	–0.125^†^	0.008	0.019	–0.152*
	–0.04,0.03	–0.07,–0.01	–0.20,0.20	–0.35,0.04	–0.26,0.01	–0.08,0.10	–0.20,0.24	–0.30,–0.00
*N*	2,823	3,035	2,833	3,034	2,908	3,118	2,873	3,072

	High Blood Pressure		Obesity		WHR **×** 10		FEV1/FVC		Metabolic Syndrome	
	Men OR/CI	Women OR/CI	Men OR/CI	Women OR/CI	Men B/CI	Women B/CI	Men B/CI	Women B/CI	Men OR/CI	Women OR/CI
Age at First Birth (ref. = 23–27 (M) / 20–24 (W))										
<23 (M) / <20 (W)	0.978	1.009	1.310*	1.003	0.091*	0.036	–0.004	–0.016*	1.216^†^	1.279^†^
	0.74,1.30	0.63,1.61	1.04,1.65	0.79,1.28	0.03,0.15	–0.03,0.10	–0.02,0.01	–0.03,–0.00	0.97,1.52	1.00,1.64
28–32 (M) / 25–29 (W)	1.060	1.136	1.017	0.725*	0.013	–0.036	–0.006	0.001	1.119	0.879
	0.83,1.35	0.79,1.63	0.83,1.25	0.59,0.89	–0.04,0.06	–0.09,0.02	–0.02,0.00	–0.01,0.01	0.93,1.35	0.71,1.08
33–38 (M) / 30–34 (W)	0.810	0.520*	0.770*	0.592*	–0.040	–0.039	–0.009	–0.005	0.776*	0.798^†^
	0.60,1.10	0.30,0.91	0.59,1.00	0.45,0.77	–0.10,0.02	–0.10,0.02	–0.02,0.00	–0.02,0.01	0.62,0.97	0.61,1.04
39+ (M) / 35+ (W)	0.801	0.798	0.818	0.671*	–0.055	–0.100*	0.006	–0.016^†^	0.766	0.694^†^
	0.48,1.33	0.40,1.57	0.53,1.26	0.47,0.97	–0.15,0.04	–0.19,–0.01	–0.01,0.03	–0.03,0.00	0.53,1.12	0.48,1.01
*N*	3,310	3,632	3,299	3,629	3,325	3,665	3,255	3,588	3,007	3,260

*Notes:* Included confounders are social class at birth, parental years of education, mother stayed at school beyond the minimum leaving age, financial hardship at age 11, overcrowding at age 11, housing conditions at age 11, birth weight, mother smoked during pregnancy, child was out of school for a month or more because of health problems, number of hospitalizations before age 11, enuresis at ages 7 and 11, poor physical coordination at age 11, mental health at ages 7 and 11, Rutter Behaviour Scales completed by the mother at ages 7 and 11, family difficulties at age 7, parental divorce before age 11, smoking at age 16, cognitive ability at age 11, need for special education at age 11, parents’ interest in the respondent’s education at age 11, ethnicity, level of education at age 23, number of partnerships by age 42, ever unemployed between January 1978 and December 2001, and age at the biomedical interview.

^†^*p* < .10; *p < .05

In sum, there was an inverse association between age at first birth and several cardiometabolic biomarkers, with worse outcomes for those with very young ages at entry to parenthood and increasingly better outcomes for older parents, especially among women.

#### *Age at Last Birth*

**Fig. 3:**
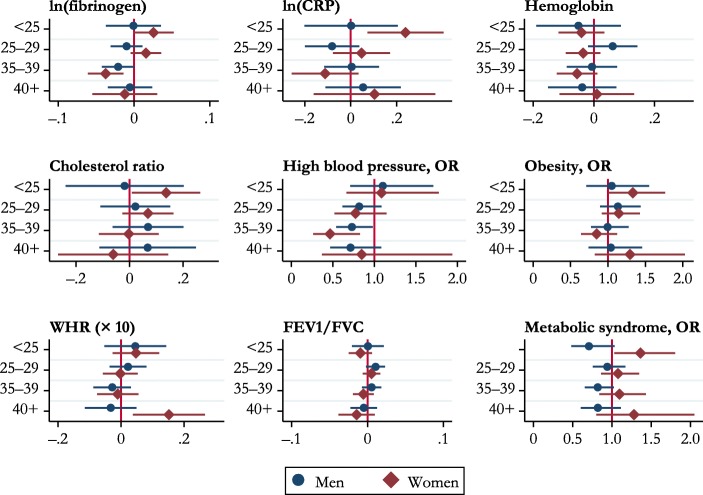
Age at last birth (ref. = 30–34) and biomarkers. Horizontal lines represent 95% confidence intervals.

The results reported in Table 6 and Fig. 3 refer to age at last birth for only those who had two or more children. Age at last birth and biomarkers are associated, although this association is less marked than for age at first birth. More specifically, for women, having the last child before age 25 was associated with a higher level of CRP (*B* = 0.239, *p* < .05), a higher cholesterol ratio (*B* = 0.136, *p* < .05), and higher risk of obesity (OR = 1.334, CI = [1.01, 1.77]). The ages at last birth that seemed to be associated with more favorable outcomes were 35–39: both men and women who had their last child at ages 35–39 had lower levels of fibrinogen (*B* = –0.021, *p* < .10 for men, and *B* = –0.037, *p* < .05 for women) and a lower probability of high blood pressure (OR = 0.729, CI = [0.54, 0.98] for men; OR = 0.467, CI = [0.26, 0.83] for women). Having the last child after age 40 was associated with a slightly WHR ratio among women (*B* = 0.152, *p* < .05).

**Table 6:** Multivariate regression models: Biomarkers and age at last birth

	log(Fibrinogen)		log(CRP)		Glycated Hemoglobin		Cholesterol Ratio (Total/HDL)	
	Men B/CI	Women B/CI	Men B/CI	Women B/CI	Men B/CI	Women B/CI	Men B/CI	Women B/CI
Age at Last Birth (ref. = 30–34)								
<25	–0.001	0.026^†^	0.002	0.239*	–0.051	–0.041	–0.019	0.136*
	–0.04,0.04	–0.00,0.05	–0.20,0.21	0.07,0.40	–0.19,0.09	–0.12,0.03	–0.24,0.20	0.01,0.26
25–29	–0.010	0.016	–0.081	0.047	0.062	–0.035	0.022	0.069
	–0.03,0.01	–0.00,0.04	–0.20,0.04	–0.08,0.17	–0.02,0.14	–0.09,0.02	–0.11,0.15	–0.03,0.16
35–39	–0.021^†^	–0.037*	0.004	–0.112	–0.006	–0.055	0.069	–0.003
	–0.04,0.00	–0.06,–0.01	–0.12,0.12	–0.26,0.03	–0.09,0.08	–0.12,0.01	–0.06,0.20	–0.12,0.11
40+	–0.005	–0.012	0.054	0.104	–0.038	0.010	0.067	–0.061
	–0.03,0.02	–0.05,0.03	–0.11,0.22	–0.16,0.37	–0.15,0.07	–0.11,0.13	–0.11,0.25	–0.27,0.14
*N*	2,197	2,501	2,205	2,500	2,259	2,565	2,240	2,530

	High Blood Pressure		Obesity		WHR **×** 10		FEV1/FVC		Metabolic Syndrome	
	Men OR/CI	Women OR/CI	Men OR/CI	Women OR/CI	Men B/CI	Women B/CI	Men B/CI	Women B/CI	Men OR/CI	Women OR/CI
Age at Last Birth (ref = 30–34)										
<25	1.101	1.087	1.051	1.334*	0.045	0.048	0.001	–0.009	0.708^†^	1.365*
	0.71,1.71	0.67,1.78	0.71,1.55	1.01,1.77	–0.05,0.14	–0.03,0.12	–0.02,0.02	0.02,0.01	0.48,1.03	1.03,1.81
25–29	0.819	0.773	1.134	1.145	0.022	–0.002	0.011^†^	0.005	0.941	1.079
	0.62,1.09	0.52,1.15	0.90,1.44	0.92,1.43	–0.04,0.08	–0.06,0.05	–0.00,0.02	–0.01,0.02	0.76,1.17	0.86,1.35
35–39	0.729*	0.467*	0.995	0.849	–0.028	–0.010	0.005	–0.005	0.820^†^	1.098
	0.54,0.98	0.26,0.83	0.78,1.28	0.64,1.12	–0.09,0.03	–0.08,0.06	–0.01,0.02	–0.02,0.01	0.66,1.03	0.84,1.43
40+	0.713	0.847	1.038	1.295	–0.033	0.152*	–0.005	–0.014	0.822	1.282
	0.47,1.08	0.37,1.94	0.74,1.46	0.83,2.03	–0.11,0.05	0.04,0.27	–0.02,0.01	–0.04,0.01	0.61,1.12	0.80,2.05
*N*	2,555	2,960	2,556	2,973	2,574	3,000	2,517	2,931	2,326	2,675

*Notes:* Included confounders are social class at birth, parental years of education, mother stayed at school beyond the minimum leaving age, financial hardship at age 11, overcrowding at age 11, housing conditions at age 11, birth weight, mother smoked during pregnancy, child was out of school for a month or more because of health problems, number of hospitalizations before age 11, enuresis at ages 7 and 11, poor physical coordination at age 11, mental health at ages 7 and 11, Rutter Behaviour Scales completed by the mother at ages 7 and 11, family difficulties at age 7, parental divorce before age 11, smoking at age 16, cognitive ability at age 11, need for special education at age 11, parents’ interest in the respondent’s education at age 11, ethnicity, level of education at age 23, number of partnerships by age 42, ever unemployed between January 1978 and December 2001, and age at the biomedical interview.

^†^*p* < .10; *p < .05

## Discussion

Previous studies have reported that both nulliparous and high-parity women (and in some studies, men) have higher risks of mortality and morbidity than parents of two or three children. In our study, indicators of cardiometabolic risk among high-parity women tended to be raised, but associations were attenuated once we controlled for a wide range of variables relating to early-life socioeconomic background, childhood physical and mental health and cognitive ability, and adult socioeconomic characteristics. However, even in the fully adjusted model, high-parity was significantly associated with high WHR and metabolic syndrome among women and with raised glycated hemoglobin among both men and women with four or more children (and remained so in analyses applying the Bonferroni correction). These findings are consistent with studies that reported an association between higher parity and obesity and glycated hemoglobin and stronger associations between higher parity and these cardiometabolic risk factors among women compared with men (Lawlor et al. 2003). We were not able, however, to formally test whether sex differences in the strength of these associations were statistically significant. Possible reasons for a differential effect on women and men might be related to changes experienced in pregnancy and also greater effects of child-rearing on the eating and physical activity patterns of mothers compared with fathers. However, this would need to be examined in a different study. Perhaps, gender differences in associations between family-building and obesity become more evident in later middle age. Hardy et al. (2007), in a study that extended to age 53, found that the difference in BMI between men without children and men with at least one child changed with increasing age with a faster increase in BMI among fathers. We included a much wider range of controls for earlier life circumstances than previous studies, thus reducing the chance that these results reflect confounding by circumstances prior to family building. Even so, some caution is needed in interpreting these results because in our robustness check analyses using negative controls, we also found an association between high parity and arm from which the blood sample was taken among women. Given that there is no conceivable mechanism that could link these variables, there may be some underlying source of bias. We found no indicators of adverse biomarker measures for the nulliparous other than some suggestion of a worse FEV1/FVC score for childless men (significant only at 10% level), which is consistent with previous studies showing worse health from smoking-related diseases in this group.

Our finding that associations between parity and the biomarkers considered were rather weaker than might have been expected from some other studies may have resulted from our controlling for a much wider range of contemporaneously collected childhood factors, including some (e.g., cognitive ability and mental health) that cannot be reliably assessed retrospectively (Jivraj et al. 2017), in addition to educational level, experience of unemployment, reported teenage smoking, and indicators of adult partnership history. However, results (reported in the online appendix) from analyses using a reduced set of covariates, similar to those included in other studies with more limited information, were very close to those from the fully adjusted models. This does not mean that the additional covariates we included are unimportant. Several, such as birth weight and physical coordination problems at age 11, were consistently associated with the biomarker outcomes considered (online appendix, Table A5). However, in other studies lacking such information, the effect of these factors may be indirectly captured via intermediary or associated covariates included.

Contextual influences may also be important. Those born in the United Kingdom in 1958 have had access to modern methods of birth control throughout their reproductive life: oral contraceptives became available in 1961, and abortion was legalized in 1967. Results from the 1976 Family Formation Survey (Dunnell 1979) showed a large increase between the mid-1960s and the mid-1970s in the proportion of higher-order births reported as planned, and it seems reasonable to assume that this proportion has subsequently increased further. Other studies that focused on earlier-born cohorts may have included a larger proportion of high-parity parents who had not made an active choice to have a large family. These parents might have experienced greater stress than the small group of high-parity parents considered here as suggested by evidence of long-term effects of unintended births on mental health (Herd et al. 2016). Age at point of observation may in itself be relevant when comparing our findings with those of other studies. Possibly some health-related sequelae of parity become evident only in later-middle or older age.

Our findings on associations between age at childbearing and biomarkers indicative of respiratory and cardiometabolic risk are more consistent with previous research. Overall results suggest an inverse relationship between age at first birth and biomarkers indicative of poorer health, including those associated with inflammation and obesity; outcomes were worse for those who had become parents at an early age compared with those who postponed entry to parenthood. Among women with two or more children, having had the last one before age 25 was also associated with some negative health indicators. In general, associations appear greater for women than for men, which is consistent with the general impact of parenthood on women’s activities and roles, although as we already noted, we were not able to formally test for gender difference. A small adverse association between late age at last motherhood (40 or older) and WHR was also observed; however, this may reflect the short interval between the last birth and the collection of the biomarker data, which may not have given some women sufficient time to return to their prepregnancy body weight.

Given the wide range of controls included in the analyses, these results are supportive of the hypothesis that stressors associated with early parenthood may have long-term health consequences. Results are partly consistent with the small literature on fertility history and biomarkers. For example, Grundy and Read (2015) found a negative association between age at first birth and higher (worse) allostatic load, with the relationship being mediated in part by wealth, physical activity, and smoking; Lacey et al. (2017) found that becoming a parent before age 20 was associated with an adverse cardiovascular profile by midlife for both men and women.

Strengths of this study include the availability of a large population-based and representative prospective study, the wealth of information on potential confounders gathered contemporaneously rather than retrospectively, and the availability of midlife biomarker data enabling us to use observer-measured indicators of health rather than relying on self-reported measures that may be influenced by health expectations and morale. Additionally, biomarker data provide some information on possible underlying mechanisms that may lead to poor health.

To explore differences that may arise using biomarkers rather than self-reported measures, we estimated models using self-rated health at the time of biomedical sweep as the outcome (see the online appendix for results). For women, associations among number of children, age at first/last birth, and poor or fair self-rated health were broadly consistent with those observed using biomarkers. Among men, however, there were some differences. For example, age at entry to fatherhood was associated with hemoglobin, obesity, and metabolic syndrome but not with fair/poor self-rated health. This may indicate that some men are not aware of, or are unwilling to report, conditions associated with poorer health outcomes and perhaps do not consider being overweight or obese as indicative of health status. This is something that merits further investigation.

We can identify five limitations of this study. First, a large proportion of data were missing because of attrition. We took account of this by using multiple imputation with chained equations (see the online appendix). We additionally performed the same set of analyses on the complete case sample (reported in the online appendix). These models produced broadly similar results in terms of point estimates, but fewer reached conventional levels of statistical significance, reflecting the much smaller sample size. Aside from the lack of power, some differences in the value of point estimates from models based on multiply imputed data and those from complete case analysis are expected because in this instance missing data can be found in the independent control variables as well as the outcome. In such instances, complete case analysis is biased (Hughes et al. 2019). Second, selection into fertility pathways is an important factor to consider, and even though we were able to include in the analysis many confounders related to childhood health and early-life conditions, it is still possible that some unobserved variables might explain the associations we found. To address this issue, we used negative controls, a technique designed to detect possible sources of spurious causal inference. We found no association between fertility histories and negative controls as expected except that (as mentioned earlier) women who had four or more children were less likely to have their blood taken from their left arm, possibly suggesting that the relationship between parity and biomarkers is partly due to confounding and/or measurement error. Third, we could not fully tackle issues of reverse causation. Particular fertility patterns may be the outcome of health problems; for example, poor health may delay fertility or decrease chances of parity progression. We included a large range of control variables capturing health in childhood and adolescence but had no specific information on fecundity. Because we did not include measures of health in adulthood, measures in this design would be mediators and not proper confounders. A fourth limitation is that although we hypothesized that earlier parenthood may have adverse implications for later-health because of the effects of cumulated stress, we do not have any measures of this collected at the time. This would be a fruitful area for further research using data from younger cohorts. Finally, the respondents in our sample were only 45 years old when the NCDS biomedical sweep was conducted. Thus, some of them (especially men) might not have yet completed their fertility, and associations between fertility history and health indicators may vary by age.

Our aim in this study was to investigate associations between fertility patterns and biomarkers indicative of cardiometabolic health in midlife, controlling for a wide range of potential confounders from earlier in life. Unlike several previous studies (e.g., Henretta 2007), we deliberately did not include in our analysis variables from later in the life course—such as other health indicators in midlife—that might be either confounders or mediators of associations with biomarkers. Examining these later pathways more thoroughly would be a fruitful line for further enquiry.

We conducted multiple tests and from a conventional null hypothesis significance testing perspective, this increases the probability of type I error. Therefore, we further evaluated our findings with the conservative Bonferroni correction, setting the significance cutoff at α / *n* = .05 / 9 = .0055, also reported in the online appendix. Even with this conservative correction, we found associations among age at first birth, cholesterol, obesity, and WHR in women. Going beyond conventional levels of statistical significance, evaluation of the pattern of associations, the magnitude of the point estimates, and pattern of *p* values points to an association between age at first birth and various biomarkers.

Results from this analysis help shed some light on the association between fertility and health and identify groups in the population that are more at-risk of health issues later in life. In particular, our findings support previous findings suggesting potential negative consequences of very early ages at first birth, which emphasizes the need for continuing policy attention to the sexual and reproductive health of young adults and the support of young parents.

## Electronic supplementary material

ESM 1 (PDF 2516 kb)

## Footnotes

1 Both fibrinogen and CRP were log-transformed to ensure a less skewed distribution.

2 In preliminary analyses, we also considered length of interbirth intervals and experience of the death of a child. However, these showed no association with any of the biomarkers (results available from the corresponding author).

3 Most of the early-life conditions and childhood health questions were asked of study members’ parents: social class at birth, parental education, mother staying in school after minimum age, financial hardship, overcrowding, housing conditions, birth weight, mother smoking during pregnancy, whether the child was out of school for more than a month, the number of times the child was hospitalized, enuresis problems, physical coordination problems, the Rutter Behaviour Scale, family difficulties and parental divorce, and parental interest in child’s education. Two of the control variables were asked of study members’ teachers: the BSAG score and the general ability score. All the other variables were asked of the main respondent.

4 The Total Syndrome scores are meant to give a quantitative assessment of the behavior pattern in question. The syndrome totals are added together to give a score that indicates, fairly crudely, the total amount of behavioral deviance (or maladjustment?) as measured by the scale’s guide. The behaviors taken into account are unforthcomingness, withdrawal, depression, anxiety for acceptance by adults, hostility toward adults, writing off of adults and adult standards, anxiety for acceptance by children, hostility toward children, restlessness, inconsequential behavior, miscellaneous symptoms, and miscellaneous nervous symptoms.

5 We include the *z* scores for psychological distress, built using an internalizing score from summing five items (worried, solitary, miserable, fearful, and fussy) and an externalizing score from summing nine items (destructive, fights, not liked, irritable, disobedient, lies, steals, bullies, and resentful/aggressive) (Winning et al. 2015).

6 Children were tested individually by teachers, who recorded the answers for the tests. For the verbal items, children were presented with an example set of four words that were linked logically, semantically, or phonologically. For the nonverbal tasks, shapes or symbols were used. The children were then given another set of three words or shapes or symbols with a blank. Participants were required to select the missing item from a list of five alternatives.

7 Given the large number of confounders, and the fact that some of them could be highly correlated (e.g., enuresis at age 7 and enuresis at age 11, general ability at age 11 and education level at age 23, and so on), we tested for multicollinearity in the complete case analysis using the variance inflation factor (VIF). For most variables, it is very close to 1; and for two variables, it is 2.2 and 3.3, respectively. In the multiple imputation analysis, using the correlation matrix, we also observed relatively low levels of correlation among the independent variables. Hence, we are not concerned with multicollinearity.

8 The tables in the text show only the linear regression coefficients/odds ratios for parity, age at first birth, and age at last birth. See the online appendix for the tables with full results. Moreover, parity is not included as a control in the regressions for age at first and last birth. As a robustness check, we also ran the models using parity as a control, and the results were very similar (available upon request). Age at first birth and age at last birth are not included in the regression models for parity so as to not exclude respondents who did not have children, and primiparous individuals are not included in the models for age at last birth because for these individuals, age at first birth and age at last birth would coincide. Some of the variables included in the regression models (e.g., education at age 23, partnership history, and employment history) could potentially be both confounders and colliders. Sensitivity analyses confirm that the results did not change when these variables were excluded. Following Greenland’s (2003) suggestion that it is better to control for a variable that is a confounder and a collider unless it is a very strong collider, we included these variables in the models reported in the text. We also corrected for multiple testing using the Bonferroni correction (we set the significance cutoff at α / *n* = .05 / 9 = .0055). We include our new sensitivity analysis in the online appendix but retain the standard analyses in the text. The Bonferroni correction could be very conservative, and we think it is also important to not overstate the role of *p* values for the validity of scientific conclusions following Waserstein 2016). We included an additional set of analysis in the online appendix, reporting results of the regression models using self-reported health as the outcome. Specifically, we created a variable of fair/poor health (= 1 if self-reported health was rated fair or poor, and 0 otherwise). The results show similar trends as for biomarkers, in particular for age at first and at last birth, with an inverted J-shaped association with the risk of reporting fair/poor health.

9 As a robustness check, we ran all the models including extra controls for the intake of medicines for cardiovascular and respiratory systems, and the results were very similar.
