# Supplementary material for: Fertility History and Biomarkers Using Prospective Data: Evidence From the 1958 National Child Development Study
Source: Demography. 2020 Mar 25;57(2):529–58. doi: 10.1007/s13524-020-00855-x (PMC7162827; doi:10.1007/s13524-020-00855-x)
Supplement: Supplementary file 1 — (PDF 2516 kb) [file 13524_2020_855_MOESM1_ESM.pdf]

Online Appendix

Multiple Imputation

We performed Multiple Imputation with chained equations with 80 imputed datasets using all variables in the substantive model, as well as auxiliary variables in the imputation process. We perform two multiple imputations: the first one on all the individuals except for those who died or migrated before age 46 (15,252), to run the regressions on number of children; the second one only on those who had information on the number of children (11,754) to run the regressions for age at first birth (on those who had at least one child) and age at last birth (on those who had at least 2 children). *We then performed our regression analysis only on those with non-missing outcomes.* Variables included in the imputation:

- Biomarkers and health variables: ln(fibrinogen), ln(c-reactive protein), glycated haemoglobin, cholesterol ratio, high blood pressure, obesity, waist-hip ratio (x10), the ratio of forced expiratory volume and forced vital capacity, metabolic syndrome, self-reported health at the biomedical survey;
- Fertility variables: number of children, age at first birth, age at last birth;
- Controls: age at interview, ethnicity, education level, number of partnerships, ever unemployed between 1978 and 2002, father's social class at birth, parents divorced at age 11, financial hardship at age 11, overcrowding at age 11, housing conditions at age 11, birth weight, mother smoking during pregnancy, Bristol Social Adjustment Guide score at age 11, parents' interest in education at age 11, teenage smoking, Rutter Behaviour Scales at age 7 and 11, number of times hospitalized at age 11, out of school for more than a month at age 11, general ability score at age 11, gender, parents' years of education, mother stayed in school after minimum leaving age, family difficulties at age 7, enuresis at age 7 and 11, physical coordination at age 11, need for special education treatment at age 11;

- Auxiliary Variables (variables associated with the biomarkers and the probability of taking part in the biomedical sweep): Currently member of Union/Staff Association, General Motor Handicap, Current activity (full-time employment, part-time employment, other), Accommodation Type (owner or not), Self-reported health at age 33, Self-reported health at age 42, Voted in the 1979 elections, Voted in the 1987 elections, Social capital at age 33.

Table A1 shows the proportion imputed for each variable used in the analysis, in the imputation for the full sample except for those who died or migrated before age 46 (15,252) and in the imputation for those who had information on the number of children (11,754). Table A2 reports the mean and the standard deviation for the biomarkers of the imputed and non-imputed samples (even though the cases with imputed biomarkers were not used in the regression analysis). Finally, Table A3 reports the estimated fraction of missing information (FMI) for the regression models for log(Fibrinogen).

Table A1 - % Imputed per Variable

	Number of Children Imputation	Age at First and Last Child Imputation
**Health Measures**		
log(Fibrinogen)	51.4	38.2
log(C-Reactive Protein)	51.3	38.1
Glycated Haemoglobin	49.9	36.4
Cholesterol Ratio (Total/HDL)	50.6	37.3
% with High Blood Pressure	41.7	26.1
% Obese	41.8	26.3
Waist to Hip Ratio	41.3	25.6
% with Metabolic Syndrome	47.6	33.6
FEV1/FVC	42.5	27.2
**Fertility Measures**		
Number of Children	22.9	0.0
Age at First Birth	36.2	17.3
Age at Last Birth	50.9	36.2
**Early Life Socioeconomic Background**		
Social Class at Birth	18.1	14.9
Financial Hardship - Age 11	21.4	17.6
Overcrowding - Age 11	18.9	15.0
Housing - Age 11	19.8	16.0
Family Difficulties - Age 7	19.2	16.3
Divorced Parents - Age 11	9.9	7.0
Mother in School after Minimum Age - Age 0	6.5	5.5
Parents' Years of Education - Age 16	31.8	27.0
Parents interested in R Education - Age 11	17.5	14.4
**Early Life Health**		

Birth Weight (ounces)	6.5	5.6
Mother smoking when pregnant	6.1	5.2
Out of school for 1+ months - Age 11	10.8	7.8
# times hospitalized - Age 11	18.7	14.9
Enuresis - Age 7	15.6	12.6
Enuresis - Age 11	18.9	15.1
Physical Coordination Problems - Age 11	19.6	16.7
BSGA Tot 'Syndrome' Score - Age 11	16.8	13.8
Rutter Scale - Age 7	15.2	12.3
Rutter Scale - Age 11	18.8	15.0
Smoking - Age 16	29.3	25.0
**Cognitive Ability**		
General ability test score - Age 11	16.9	13.7
Special Education Treatment - Age 11	24.9	21.2
Education Level - Age 23	33.6	18.9
**Sociodemographic Characteristics**		
Age at Interview - Biomed Sweep	40.8	25.0
Ethnicity	5.4	0.7
# Partnerships	10.9	0.0
Ever Unemployed (1978-2001)	11.3	0.4
Total N	15,252	11,754

Table A2 - Biomarkers Mean and Standard Deviation - Imputed vs Non-imputed

	**Number of Children Imputation**				**Age at First and Last Child Imputation**			
	**Non-Imputed**		**Imputed**		**Non-Imputed**		**Imputed**	
	Mean	SD	Mean	SD	Mean	SD	Mean	SD
Fibrinogen (g/L)	2.96	0.62	2.97	0.46	2.96	0.62	2.96	0.51
C-Reactive Protein (g/L)	2.19	4.34	1.76	3.14	2.19	4.36	1.88	3.52
Glycated Haemoglobin	5.26	0.70	5.30	0.53	5.25	0.70	5.28	0.58
Cholesterol Ratio (Total/HDL)	3.98	1.18	4.05	0.92	3.98	1.18	4.01	1.00
% with High Blood Pressure	10.86		11.89		10.75		11.08	
% Obese	24.60		26.59		24.51		25.83	
Waist to Hip Ratio	0.87	0.09	0.88	0.08	0.87	0.09	0.87	0.08
% with Metabolic Syndrome	35.87		40.47		35.84		39.19	
FEV1/FVC	0.78	0.12	0.78	0.09	0.78	0.12	0.78	0.10
Total N	15,252				11,754			

Table A3 - Estimated Fraction of Missing Information (FMI), log(Fibrinogen) regression models

		**log(Fibrinogen)**			**log(Fibrinogen)**			**log(Fibrinogen)**	
**Number of Children, Ref: 2 Children**		**Men**	**Women**	**Age at 1st Birth, Ref: 23-27 (M)/20-24 (W)**	**Men**	**Women**	**Age at Last Birth, Ref: 30-34**	**Men**	**Women**
	0	0.03	0.02	<23(M)-<20(W)	0.01	0.01	<25	0.01	0.01
	1	0.04	0.02	28-32 (M) / 25-29 (W)	0.01	0.01	25-29	0.01	0.01
	3	0.03	0.02	33-38 (M) / 30-34 (W)	0.01	0.01	35-39	0.01	0.01
	4	0.04	0.02	39+ (M) / 35+ (W)	0.01	0.01	40+	0.01	0.01
Age at Interview		0.01	0.01		0.01	0.01		0.01	0.01

Education (Ref: Low)								
Medium	0.22	0.15		0.17	0.13		0.15	0.10
High	0.20	0.16		0.14	0.15		0.13	0.12
Number of Partnerships (Ref: 0)								
1	0.02	0.02		0.01	0.01		0.05	0.01
2+	0.02	0.02		0.01	0.01		0.05	0.01
Ever Unemployed (1978-2001)	0.01	0.01		0.01	0.01		0.01	0.01
Social Class at Birth - Manual	0.16	0.16		0.16	0.16		0.14	0.17
Ethnicity (White vs Non-White)	0.02	0.02		0.02	0.02		0.02	0.02
Special Educ. Treatment - Age 11	0.30	0.38		0.28	0.31		0.26	0.30
Financial Hardship - Age 11	0.18	0.21		0.17	0.19		0.15	0.20
Overcrowding - Age 11	0.17	0.15		0.19	0.11		0.17	0.11
Housing Index (No access to…) - Age 11	0.16	0.19		0.19	0.16		0.20	0.17
Birth Weight	0.08	0.04		0.06	0.06		0.06	0.05
Mother smoked during pregnancy	0.05	0.05		0.06	0.05		0.06	0.05
BSGA Score - Age 11	0.14	0.13		0.16	0.21		0.18	0.20
Parents interested in Edu - Age 11	0.15	0.16		0.18	0.15		0.16	0.15
Teenage Smoking - Age 16	0.27	0.19		0.21	0.21		0.21	0.19
Rutter - Age 7	0.15	0.14		0.11	0.12		0.12	0.12
Rutter - Age 11	0.16	0.12		0.13	0.13		0.12	0.14
Parents' Years of Educ.	0.29	0.23		0.33	0.24		0.29	0.19
Mother stayd in school after min. age	0.14	0.10		0.12	0.12		0.10	0.09
Family Difficulties - Age 7	0.35	0.23		0.38	0.26		0.39	0.27
Parents Divorced by age 10/11	0.22	0.15		0.21	0.14		0.22	0.13
Times hospitalized - Age 11	0.13	0.12		0.14	0.15		0.15	0.15
Out of school for 1+ months - Age 11	0.10	0.11		0.09	0.09		0.09	0.11
General Ability - Age 11	0.19	0.17		0.13	0.19		0.15	0.18
Enuresis - Age 7	0.19	0.09		0.22	0.14		0.18	0.14
Enuresis - Age 11	0.18	0.16		0.24	0.13		0.22	0.13
Physical Coordination Problems - Age 11	0.20	0.20		0.18	0.14		0.19	0.15
Constant	0.01	0.01		0.01	0.01		0.02	0.01
N	3,699	3,713		2,823	3,035		2,197	2,501

Table A4. Descriptive information for the Control Variables

	Men				Women			
	**Mean or %**	**SD**	**N**	**N in the Sweep**	**Mean or %**	**SD**	**N**	**N in the Sweep**
**Early Life Socioeconomic Background**								
Social Class at Birth - % Manual	68.2		7,227	*9,004*	68.1		6,887	*8,411*
% in Financial Hardship - Age 11	11.1		6,857	*7,887*	11.6		6,506	*7,450*
% Overcrowding - Age 11	12.0		7,065	*7,887*	12.1		6,727	*7,450*
Housing: % NO Access to 1+ (Bathroom; Indoor WC; Cooking Facilities; Hot water) - Age 11	10.82		6,989	*7,887*	10.9		6,640	*7,450*
% with Family Difficulties - Age 7	4.46		7,152	*7,917*	4.03		6,794	*7,508*
% with Divorced Parents - Age 11	4.53		7,886	*7,887*	4.66		7,450	*7,450*
% Mother in School after Minimum Age - Age 0	24.8		8,970	*9,004*	25.1		8,383	*8,411*
Parents' Years of Education - Age 16	11.3	(1.81)	5,901	*7,547*	11.4	(1.84)	5,623	*7,107*
% Parents interested in R Education - Age 11	76.1		7,206	*7,887*	77.9		6,830	*7,450*
**Early Life Health**								
Birth Weight (ounces)	119.0	(22.0)	8,959	*9,004*	114.1	(21.1)	8,382	*8,411*
% Mother smoking when pregnant	33.2		9,004	*9,004*	33.2		8,411	*8,411*
% Out of school for 1+ months - Age 11	4.88		7,803	*7,887*	5.51		7,370	*7,450*
# times hospitalized - Age 11	0.74	(0.98)	7,089	*7,887*	0.57	(0.85)	6,736	*7,450*
% with Enuresis - Age 7	14.0		7,467	*7,917*	11.6		7,074	*7,508*
% with Enuresis - Age 11	7.37		7,069	*7,887*	4.73		6,723	*7,450*
Physical Coordination Problems - Age 11	17.2		7,028	*7,887*	13.5		6,624	*7,450*
BSGA Tot 'Syndrome' Score - Age 11	9.88	(9.67)	7,273	*7,887*	7.03	(7.95)	6,883	*7,450*
Rutter Scale - Age 7	-0.01	(1.00)	7,506	*7,917*	0.01	(1.00)	7,102	*7,508*
Rutter Scale - Age 11	0.06	(1.01)	7,078	*7,887*	-0.07	(0.98)	6,727	*7,450*
% Smoking - Age 16	37.5		6,114	*7,547*	34.0		5,855	*7,107*
**Cognitive Ability**								
General ability test score - Age 11	41.8	(16.3)	7,253	*7,887*	44.1	(15.9)	6,878	*7,450*
% need Special Education Treatment - Age 11	4.2		6,543	*7,780*	2.3		6,218	*7,516*
Education Level - Age 23								
% Low	25.5		5,205	*6,267*	29.8		5,457	*6,270*
% Medium	56.0				50.7			
% High	18.5				19.5			
**Sociodemographic Characteristics**								
Age at Interview - Biomed Sweep	45.2	(0.39)	4,659	*4,665*	45.2	(0.39)	4,709	*4,712*
Ethnicity - % White	96.6		8,257	*9,004*	96.9		7,867	*8,411*
# Partnerships - Age Biomedical Sweep	1.69	(1.32)	6,863	*7,780*	1.72	(1.29)	6,761	*7,516*
% Ever Unemployed (1978-2001)	36.5		7,439	*7,780*	25.2		7,256	*7,516*

Figure A1. Number of Children and Biomarkers (No controls)

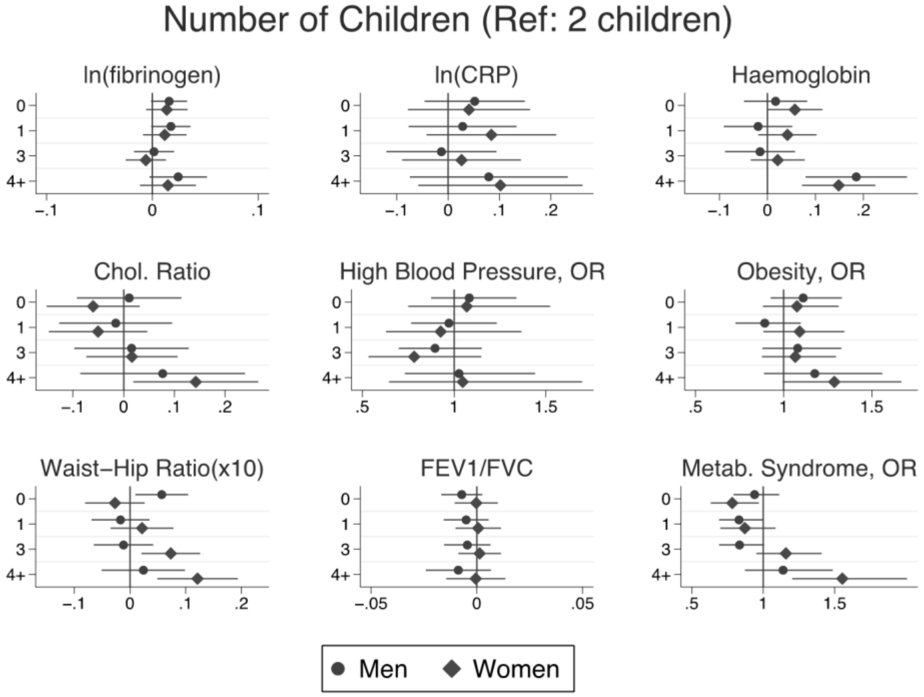

Figure A2. Number of Children and Biomarkers (Restricted set of confounders: father's social class at birth, parents divorced at age 11, overcrowding at age 11, housing conditions at age 11, teenage smoking, number of times hospitalized at age 11, out of school for more than a month at age 11, parents' years of education, education level, number of partnerships, ever unemployed between 1978 and 2002**)**

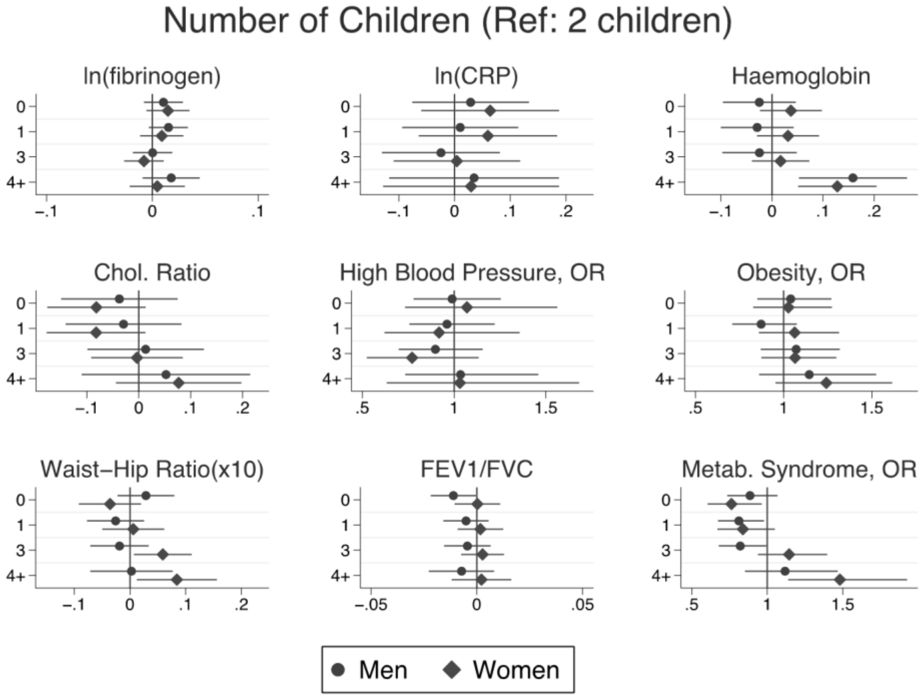

Table A5. Biomarkers and Number of Children

	log(Fibrinogen)		log(C-Reactive Protein)		Glycated Haemoglobin	
	**Men**	**Women**	**Men**	**Women**	**Men**	**Women**
**Number of Children, Ref: 2 Children**	*B/CI*	*B/CI*	*B/CI*	*B/CI*	*B/CI*	*B/CI*
0	0.009	0.016	0.029	0.067	-0.021	0.038
	-0.01,0.03	-0.00,0.04	-0.08,0.13	-0.06,0.19	-0.09,0.05	-0.02,0.10
1	0.012	0.008	-0.003	0.051	-0.035	0.032
	-0.01,0.03	-0.01,0.03	-0.11,0.10	-0.07,0.17	-0.11,0.04	-0.03,0.09
3	-0.001	-0.01	-0.026	-0.011	-0.025	0.007
	-0.02,0.02	-0.03,0.01	-0.13,0.08	-0.12,0.10	-0.10,0.05	-0.05,0.06
4+	0.013	0.005	0.01	0.031	0.123**	0.129***
	-0.01,0.04	-0.02,0.03	-0.14,0.16	-0.13,0.19	0.02,0.23	0.05,0.21
Age at Interview	-0.006	-0.015*	-0.041	-0.017	-0.130***	-0.116***
	-0.02,0.01	-0.03,0.00	-0.13,0.05	-0.12,0.09	-0.19,-0.07	-0.17,-0.06
Education (Ref: Low)						
Medium	-0.018*	-0.031***	-0.122**	-0.180***	-0.036	-0.055*
	-0.04,0.00	-0.05,-0.01	-0.23,-0.01	-0.30,-0.06	-0.12,0.04	-0.11,0.00
High	-0.039***	-0.028**	-0.254***	-0.268***	0	-0.061
	-0.06,-0.01	-0.05,-0.00	-0.40,-0.11	-0.42,-0.11	-0.10,0.10	-0.14,0.01
Number of Partnerships (Ref: 0)						
1	-0.020	-0.015	-0.125	-0.006	-0.180***	-0.105*
	-0.05,0.01	-0.05,0.02	-0.30,0.05	-0.24,0.23	-0.30,-0.06	-0.22,0.01
2+	-0.022	-0.040**	-0.096	-0.060	-0.155***	-0.127**
	-0.05,0.01	-0.08,-0.00	-0.27,0.08	-0.29,0.17	-0.27,-0.04	-0.24,-0.01
Ever Unemployed (1978-2001)	0.003	0.010	-0.011	0.008	0.020	0.041*
	-0.01,0.02	-0.01,0.03	-0.09,0.07	-0.09,0.10	-0.03,0.07	-0.00,0.09
Social Class at Birth - Manual	0.000	0.018*	0.036	0.196***	-0.075**	0.040
	-0.02,0.02	-0.00,0.04	-0.06,0.13	0.09,0.30	-0.14,-0.01	-0.01,0.09
Ethnicity (White vs Non-White)	-0.008	0.009	-0.236*	-0.013	-0.350***	-0.157**
	-0.05,0.04	-0.04,0.06	-0.49,0.02	-0.32,0.30	-0.52,-0.18	-0.31,-0.01
Special Educ. Treatment - Age 11	-0.057**	-0.026	-0.233	-0.495**	0.040	-0.514**
	-0.11,-0.00	-0.10,0.05	-0.54,0.07	-0.94,-0.05	-0.17,0.24	-0.90,-0.12
Financial Hardship - Age 11	0.013	0.023*	-0.052	0.112	-0.023	0.011
	-0.01,0.04	-0.00,0.05	-0.19,0.09	-0.04,0.27	-0.12,0.08	-0.07,0.09
Overcrowding - Age 11	-0.004	-0.001	0.014	-0.020	0.003	-0.013
	-0.03,0.02	-0.03,0.02	-0.11,0.14	-0.17,0.13	-0.09,0.09	-0.09,0.06
Housing Index (No access to…) - Age 11	0.002	0.005	0.109	-0.007	0.122**	0.038
	-0.02,0.03	-0.02,0.03	-0.03,0.24	-0.15,0.14	0.03,0.22	-0.04,0.11
Birth Weight	0.000	-0.000*	-0.001	-0.001	0.000	-0.001
	-0.00,0.00	-0.00,0.00	-0.00,0.00	-0.00,0.00	-0.00,0.00	-0.00,0.00
Mother smoked during pregnancy	0.007	-0.016**	0.093**	-0.108**	0.035	0.001
	-0.01,0.02	-0.03,-0.00	0.01,0.17	-0.20,-0.02	-0.02,0.09	-0.04,0.05

BSGA Score - Age 11	0.000	0.000	-0.002	-0.001	0.004**	0.002
	-0.00,0.00	-0.00,0.00	-0.01,0.00	-0.01,0.01	0.00,0.01	-0.00,0.01
Parents interested in Edu - Age 11	-0.014	-0.018*	-0.075	-0.119**	-0.076**	-0.016
	-0.03,0.00	-0.04,0.00	-0.18,0.03	-0.24,-0.00	-0.14,-0.01	-0.07,0.04
Teenage Smoking - Age 16	0.043***	0.024***	0.251***	0.115**	0.065**	0.059**
	0.03,0.06	0.01,0.04	0.16,0.34	0.02,0.21	0.00,0.12	0.01,0.11
Rutter - Age 7	-0.001	0.003	-0.017	0.023	-0.024*	-0.007
	-0.01,0.01	-0.01,0.01	-0.06,0.02	-0.02,0.07	-0.05,0.00	-0.03,0.02
Rutter - Age 11	0.002	0.007	0.026	0.045*	0.012	0.012
	-0.01,0.01	-0.00,0.01	-0.02,0.07	-0.00,0.10	-0.02,0.04	-0.01,0.04
Parents' Years of Educ.	-0.005*	-0.004	-0.015	-0.008	-0.017*	-0.003
	-0.01,0.00	-0.01,0.00	-0.04,0.01	-0.04,0.03	-0.03,0.00	-0.02,0.01
Mother stayd in school after min. age	0.010	-0.023**	0.014	-0.171***	-0.005	-0.025
	-0.01,0.03	-0.04,-0.00	-0.09,0.12	-0.29,-0.05	-0.07,0.06	-0.08,0.03
Family Difficulties - Age 7	0.017	0.035	0.278*	0.026	0.229**	-0.114*
	-0.03,0.07	-0.01,0.08	-0.01,0.57	-0.25,0.30	0.01,0.44	-0.24,0.02
Parents Divorced by age 10/11	-0.023	-0.036*	-0.101	-0.031	-0.103	0.078
	-0.07,0.03	-0.08,0.01	-0.37,0.17	-0.29,0.23	-0.29,0.09	-0.04,0.20
Times hospitalized - Age 11	0.004	0.007	0.036*	0.046*	0.010	-0.014
	-0.00,0.01	-0.00,0.02	-0.00,0.08	-0.01,0.10	-0.02,0.04	-0.04,0.01
Out of school for 1+ months - Age 11	0.029*	0.013	0.093	0.024	-0.004	0.014
	-0.00,0.06	-0.02,0.05	-0.08,0.27	-0.18,0.23	-0.12,0.11	-0.08,0.11
General Ability - Age 11	-0.000*	-0.001**	-0.003	-0.004*	-0.003***	0.000
	-0.00,0.00	-0.00,-0.00	-0.01,0.00	-0.01,0.00	-0.00,-0.00	-0.00,0.00
Enuresis - Age 7	0.012	-0.004	0.077	-0.083	-0.003	-0.012
	-0.01,0.03	-0.03,0.02	-0.05,0.20	-0.23,0.07	-0.09,0.08	-0.08,0.06
Enuresis - Age 11	-0.004	0.016	0.017	0.048	0.047	-0.011
	-0.03,0.03	-0.02,0.05	-0.16,0.19	-0.18,0.27	-0.08,0.17	-0.12,0.10
Physical Coordination Problems - Age 11	0.021**	0.047***	0.106*	0.251***	0.031	0.137***
	0.00,0.04	0.02,0.07	-0.01,0.22	0.10,0.40	-0.04,0.11	0.06,0.21
Constant	1.499***	1.922***	2.769	1.835	12.018***	11.240***
	0.76,2.24	1.13,2.71	-1.41,6.95	-2.94,6.61	9.19,14.85	8.86,13.62
N	3,699	3,713	3,709	3,712	3,824	3,820

* p<0.10, ** p<0.05, *** p<0.01

Table A5 Cont. Biomarkers and Number of Children

	**Cholesterol Ratio (Tot./HDL)**		**High Blood Pressure**		**Obesity**	
	**Men**	**Women**	**Men**	**Women**	**Men**	**Women**
**Number of Children, Ref: 2 Children**	*B/CI*	*B/CI*	*OR/CI*	*OR/CI*	*OR/CI*	*OR/CI*
0	-0.046	-0.077	1.006	1.092	1.044	1.037
	-0.16,0.07	-0.17,0.02	0.79,1.28	0.74,1.60	0.85,1.28	0.83,1.29
1	-0.036	-0.080*	0.97	0.95	0.866	1.056
	-0.15,0.08	-0.18,0.02	0.76,1.23	0.64,1.41	0.70,1.07	0.85,1.31

3	0.009	-0.007	0.904	0.762	1.082	1.053
	-0.10,0.12	-0.09,0.08	0.70,1.16	0.52,1.12	0.88,1.34	0.86,1.29
4+	0.039	0.083	1.049	1.072	1.125	1.237
	-0.13,0.20	-0.04,0.20	0.74,1.49	0.65,1.76	0.84,1.50	0.95,1.62
Age at Interview	0.02	-0.005	0.839	1.222	0.949	1.067
	-0.08,0.12	-0.09,0.08	0.68,1.03	0.88,1.70	0.79,1.14	0.89,1.28
Education (Ref: Low)						
Medium	-0.081	-0.169***	1.047	0.942	0.983	0.875
	-0.20,0.04	-0.26,-0.08	0.81,1.35	0.65,1.37	0.80,1.21	0.72,1.07
High	-0.108	-0.158***	1.003	0.881	0.878	0.867
	-0.27,0.05	-0.28,-0.04	0.71,1.42	0.53,1.47	0.66,1.17	0.66,1.14
Number of Partnerships (Ref: 0)						
1	-0.138	-0.247***	0.675**	1.079	0.869	0.796
	-0.33,0.05	-0.43,-0.07	0.47,0.97	0.55,2.13	0.63,1.20	0.55,1.15
2+	-0.174*	-0.296***	0.683**	0.978	0.805	0.675**
	-0.36,0.01	-0.47,-0.12	0.48,0.98	0.50,1.93	0.59,1.10	0.47,0.97
Ever Unemployed (1978-2001)	0.044	0.072*	0.96	0.913	0.975	1.105
	-0.04,0.13	-0.00,0.14	0.81,1.14	0.68,1.23	0.84,1.13	0.94,1.30
Social Class at Birth - Manual	0.082	0.155***	1.287**	1.512**	1.178*	1.294***
	-0.02,0.18	0.07,0.24	1.03,1.60	1.05,2.18	0.98,1.42	1.07,1.57
Ethnicity (White vs Non-White)	0.034	0.157	0.895	1.042	1.095	0.783
	-0.23,0.30	-0.07,0.39	0.53,1.52	0.43,2.52	0.67,1.79	0.48,1.27
Special Educ. Treatment - Age 11	-0.106	-0.301	1.907*	0.565	0.862	0.669
	-0.42,0.20	-0.67,0.06	0.94,3.88	0.19,1.70	0.52,1.44	0.36,1.25
Financial Hardship - Age 11	0.005	0.064	1.194	0.703	0.942	1.024
	-0.15,0.16	-0.05,0.18	0.88,1.62	0.42,1.18	0.72,1.24	0.79,1.33
Overcrowding - Age 11	-0.022	0.023	0.924	1.186	0.951	0.792*
	-0.16,0.12	-0.09,0.14	0.69,1.24	0.76,1.85	0.74,1.23	0.62,1.02
Housing Index (No access to…) - Age 11	-0.276***	0.031	1.188	0.819	0.842	1.087
	-0.42,-0.13	-0.09,0.15	0.90,1.57	0.51,1.32	0.65,1.10	0.85,1.38
Birth Weight	0	-0.002*	0.997	0.985***	1.006***	1.004**
	-0.00,0.00	-0.00,0.00	0.99,1.00	0.98,0.99	1.00,1.01	1.00,1.01
Mother smoked during pregnancy	-0.019	0.034	1.014	0.999	1.377***	1.182**
	-0.10,0.07	-0.04,0.10	0.85,1.22	0.75,1.33	1.18,1.61	1.01,1.38
BSGA Score - Age 11	0.001	0.001	0.996	0.991	1.006	1
	-0.00,0.01	-0.00,0.01	0.98,1.01	0.97,1.01	1.00,1.02	0.99,1.01
Parents interested in Edu - Age 11	-0.055	-0.038	0.961	1.077	0.915	0.902
	-0.17,0.06	-0.13,0.05	0.76,1.21	0.75,1.56	0.75,1.12	0.74,1.10
Teenage Smoking - Age 16	0.130***	0.171***	0.926	1.035	0.93	0.888
	0.04,0.22	0.10,0.25	0.76,1.13	0.75,1.43	0.78,1.11	0.75,1.06
Rutter - Age 7	-0.033	-0.002	0.944	1.081	0.928*	1.061
	-0.08,0.01	-0.04,0.04	0.86,1.04	0.93,1.26	0.85,1.01	0.98,1.15

Rutter - Age 11	0.067***	0.054***	1.026	1.056	1.077*	1.087*
	0.02,0.11	0.02,0.09	0.93,1.13	0.90,1.24	0.99,1.17	1.00,1.19
Parents' Years of Educ.	-0.032**	-0.023*	0.945	1.053	0.945*	0.978
	-0.06,-0.00	-0.05,0.00	0.88,1.02	0.95,1.17	0.89,1.00	0.92,1.04
Mother stayd in school after min. age	0.065	-0.092*	1.089	0.957	0.851	0.849
	-0.04,0.17	-0.19,0.00	0.85,1.39	0.65,1.41	0.69,1.05	0.68,1.06
Family Difficulties - Age 7	-0.056	0.113	1.22	0.502	1.4	1.038
	-0.37,0.26	-0.11,0.34	0.67,2.24	0.18,1.44	0.82,2.40	0.64,1.68
Parents Divorced by age 10/11	0.184	-0.117	0.797	1.397	1.024	1.009
	-0.13,0.49	-0.31,0.08	0.44,1.45	0.64,3.05	0.61,1.71	0.66,1.55
Times hospitalized - Age 11	-0.019	0.011	0.974	0.903	0.986	1.086*
	-0.06,0.02	-0.03,0.05	0.89,1.07	0.76,1.07	0.91,1.07	0.99,1.19
Out of school for 1+ months - Age 11	0.152	-0.016	1.093	0.892	1.278	1.042
	-0.04,0.34	-0.17,0.13	0.74,1.61	0.47,1.69	0.93,1.76	0.75,1.44
General Ability - Age 11	-0.001	-0.001	0.999	1.003	0.996	0.996
	-0.00,0.00	-0.00,0.00	0.99,1.01	0.99,1.01	0.99,1.00	0.99,1.00
Enuresis - Age 7	-0.038	0.026	0.964	1.179	0.837	0.986
	-0.17,0.09	-0.09,0.14	0.73,1.28	0.77,1.81	0.65,1.07	0.77,1.27
Enuresis - Age 11	0.095	-0.035	1.059	1.399	1.251	1.072
	-0.09,0.27	-0.21,0.14	0.73,1.53	0.77,2.56	0.91,1.72	0.74,1.56
Physical Coordination Problems - Age 11	0.104	0.205***	1.047	1.274	1.095	1.564***
	-0.02,0.23	0.09,0.31	0.82,1.34	0.85,1.91	0.88,1.36	1.24,1.97
Constant	4.117*	4.642**	1127.216	0	4.319	0.034
	-0.36,8.59	0.95,8.33	0.08,1.53e+07	0.00,111.16	0.00,14832.04	0.00,137.05
N	3,770	3,766	4,424	4,470	4,405	4,473

* p<0.10, ** p<0.05, *** p<0.01

Table A5 Cont. Biomarkers and Number of Children

	**Waist-Hip Ratio*10**		**FEV1/FVC**		**Metabolic Syndrome**	
	**Men**	**Women**	**Men**	**Women**	**Men**	**Women**
**Number of Children, Ref: 2 Children**	*B/CI*	*B/CI*	*B/CI*	*B/CI*	*OR/CI*	*OR/CI*
0	0.019	-0.031	-0.011**	0.001	0.877	0.780**
	-0.03,0.07	-0.09,0.02	-0.02,-0.00	-0.01,0.01	0.73,1.06	0.62,0.99
1	-0.033	0.005	-0.005	0.002	0.800**	0.839
	-0.08,0.02	-0.05,0.06	-0.02,0.01	-0.01,0.01	0.66,0.97	0.67,1.05
3	-0.019	0.054**	-0.005	0.003	0.819**	1.13
	-0.07,0.03	0.00,0.11	-0.02,0.01	-0.01,0.01	0.68,0.99	0.92,1.38
4+	-0.011	0.078**	-0.009	0.003	1.096	1.524***
	-0.08,0.06	0.01,0.15	-0.02,0.01	-0.01,0.02	0.83,1.44	1.17,1.99
Age at Interview	0.003	0.075***	0	-0.005	0.883	0.981
	-0.04,0.05	0.03,0.12	-0.01,0.01	-0.01,0.00	0.75,1.04	0.81,1.18
Education (Ref: Low)						
Medium	-0.093***	-0.068**	0.005	0.006	0.853	0.945
	-0.15,-0.04	-0.12,-0.01	-0.01,0.02	-0.00,0.02	0.70,1.04	0.77,1.16
High	-0.132***	-0.068*	0.008	-0.004	0.877	0.888

	-0.21,-0.06	-0.14,0.00	-0.01,0.02	-0.02,0.01	0.67,1.14	0.67,1.18
Number of Partnerships (Ref: 0)						
1	-0.071*	-0.085*	-0.013	0.011	0.796	0.82
	-0.15,0.01	-0.19,0.02	-0.03,0.01	-0.01,0.03	0.58,1.09	0.54,1.23
2+	-0.079*	-0.08	-0.012	0.005	0.813	0.723
	-0.16,0.00	-0.18,0.02	-0.03,0.01	-0.01,0.02	0.60,1.11	0.48,1.09
Ever Unemployed (1978-2001)	-0.003	0.01	-0.001	0.002	1.022	1.108
	-0.04,0.03	-0.03,0.05	-0.01,0.01	-0.01,0.01	0.89,1.17	0.94,1.31
Social Class at Birth - Manual	0.019	0.064***	0.005	0.003	1.009	1.578***
	-0.03,0.06	0.02,0.11	-0.00,0.01	-0.01,0.01	0.85,1.19	1.29,1.94
Ethnicity (White vs Non-White)	0.066	0.031	-0.011	-0.003	1.033	1.134
	-0.05,0.18	-0.10,0.16	-0.04,0.01	-0.03,0.02	0.66,1.61	0.67,1.92
Special Educ. Treatment - Age 11	-0.042	-0.327***	-0.013	-0.016	0.988	0.789
	-0.18,0.10	-0.52,-0.13	-0.04,0.02	-0.05,0.02	0.58,1.68	0.38,1.63
Financial Hardship - Age 11	-0.059*	0.046	-0.001	-0.012*	0.782*	0.917
	-0.13,0.01	-0.02,0.12	-0.02,0.01	-0.03,0.00	0.60,1.02	0.70,1.20
Overcrowding - Age 11	0	0.028	-0.004	0.006	0.782**	1.048
	-0.06,0.06	-0.04,0.09	-0.02,0.01	-0.01,0.02	0.62,0.99	0.82,1.34
Housing Index (No access to…) - Age 11	0.017	0.058*	-0.003	0.006	0.954	1.065
	-0.05,0.08	-0.01,0.12	-0.02,0.01	-0.01,0.02	0.75,1.21	0.83,1.37
Birth Weight	0.001*	0.001	0	0	1.004**	0.997
	-0.00,0.00	-0.00,0.00	-0.00,0.00	-0.00,0.00	1.00,1.01	0.99,1.00
Mother smoked during pregnancy	0.058***	0.057***	-0.007*	-0.007*	1.109	0.978
	0.02,0.10	0.02,0.10	-0.02,0.00	-0.02,0.00	0.96,1.28	0.83,1.15
BSGA Score - Age 11	0.002*	0	0	0	1	0.986**
	-0.00,0.00	-0.00,0.00	-0.00,0.00	-0.00,0.00	0.99,1.01	0.97,1.00
Parents interested in Edu - Age 11	-0.01	-0.029	0.003	0.002	0.91	0.831*
	-0.06,0.04	-0.08,0.02	-0.01,0.01	-0.01,0.01	0.75,1.10	0.68,1.01
Teenage Smoking - Age 16	0.054**	0.084***	-0.015***	-0.012***	1.156*	1.159
	0.01,0.10	0.04,0.13	-0.02,-0.01	-0.02,-0.00	0.99,1.36	0.97,1.39
Rutter - Age 7	-0.011	0.003	-0.001	0.001	0.910**	1.053
	-0.03,0.01	-0.02,0.02	-0.01,0.00	-0.00,0.01	0.84,0.98	0.97,1.15
Rutter - Age 11	0.014	0.017	-0.004*	-0.001	1.085**	1.012
	-0.01,0.04	-0.01,0.04	-0.01,0.00	-0.01,0.00	1.00,1.17	0.92,1.11
Parents' Years of Educ.	-0.020***	-0.008	-0.002	0.001	0.937**	0.977
	-0.03,-0.01	-0.02,0.01	-0.00,0.00	-0.00,0.00	0.89,0.99	0.92,1.04
Mother stayd in school after min. age	-0.022	0.015	0.005	-0.003	1.027	0.908
	-0.07,0.03	-0.04,0.07	-0.01,0.02	-0.01,0.01	0.85,1.24	0.72,1.14
Family Difficulties - Age 7	-0.004	-0.008	0.003	0.013	1.109	1.154
	-0.15,0.14	-0.13,0.12	-0.03,0.03	-0.01,0.04	0.68,1.81	0.72,1.85

Parents Divorced by age 10/11	-0.042	-0.041	-0.008	-0.020*	0.933	0.988
	-0.17,0.09	-0.16,0.07	-0.04,0.02	-0.04,0.00	0.58,1.49	0.64,1.53
Times hospitalized - Age 11	-0.003	0.014	-0.001	-0.005*	0.959	1.059
	-0.02,0.02	-0.01,0.04	-0.01,0.00	-0.01,0.00	0.89,1.03	0.97,1.16
Out of school for 1+ months - Age 11	0.097**	0.008	-0.007	0.01	0.921	1.168
	0.01,0.18	-0.08,0.10	-0.03,0.01	-0.01,0.03	0.67,1.26	0.83,1.64
General Ability - Age 11	-0.003***	-0.002**	0	0	0.994**	0.992**
	-0.00,-0.00	-0.00,-0.00	-0.00,0.00	-0.00,0.00	0.99,1.00	0.99,1.00
Enuresis - Age 7	-0.038	0.016	-0.005	0.018***	0.972	1.097
	-0.10,0.02	-0.05,0.08	-0.02,0.01	0.01,0.03	0.78,1.21	0.85,1.42
Enuresis - Age 11	0.04	0.055	0.015*	-0.027***	1.167	0.909
	-0.04,0.12	-0.05,0.16	-0.00,0.03	-0.05,-0.01	0.86,1.58	0.62,1.34
Physical Coordination Problems - Age 11	0.128***	0.139***	-0.002	0.01	1.224*	1.685***
	0.07,0.18	0.07,0.20	-0.01,0.01	-0.00,0.02	0.99,1.51	1.33,2.14
Constant	9.471***	5.098***	0.830***	1.013***	565.699*	2.631
	7.45,11.49	2.96,7.23	0.40,1.26	0.60,1.43	0.32,1.01e+06	0.00,14074.73
N	4,444	4,517	4,343	4,421	3,980	4,011

* p<0.10, ** p<0.05, *** p<0.01

Figure A3. Age at First Birth and Biomarkers (No controls)

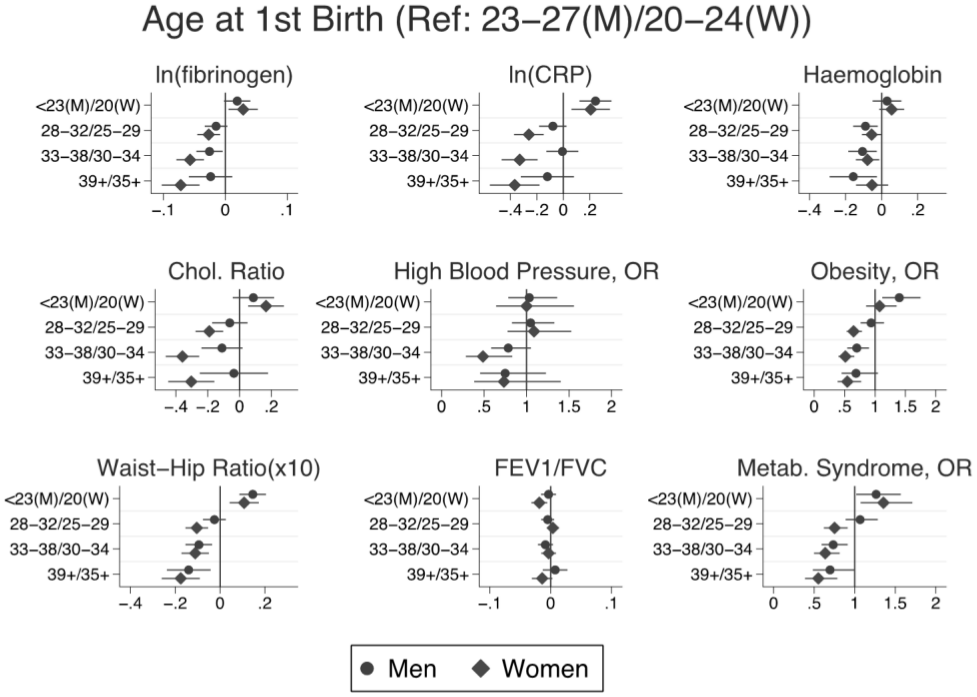

Figure A4. Age at First Birth and Biomarkers (Restricted set of confounders: father's social class at birth, parents divorced at age 11, overcrowding at age 11, housing conditions at age 11, teenage smoking, number of times hospitalized at age 11, out of school for more than a month at age 11, parents' years of education, education level, number of partnerships, ever unemployed between 1978 and 2002**)**

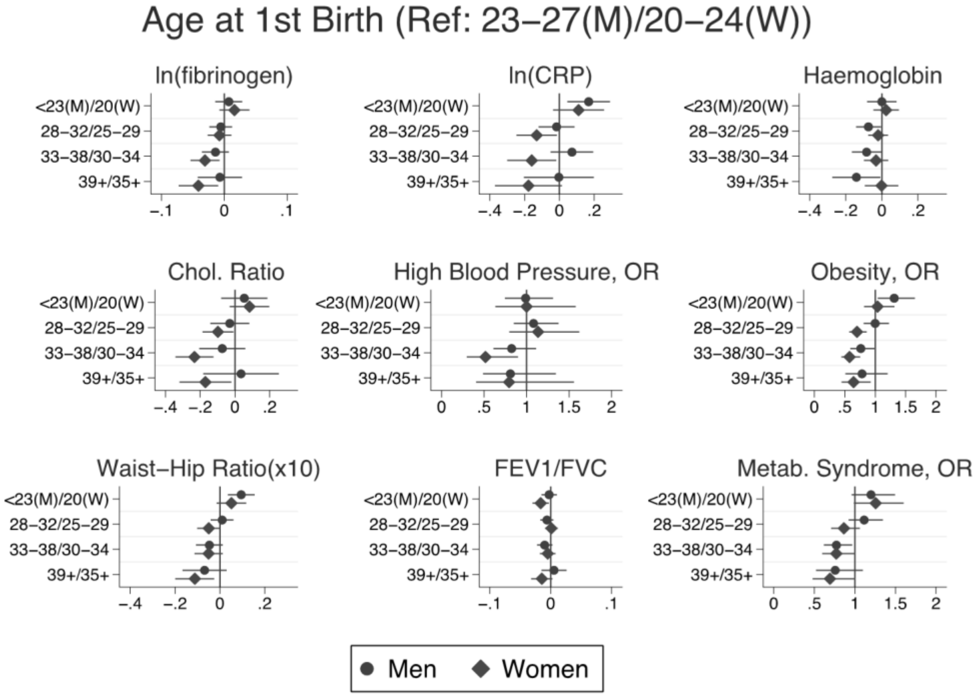

Table A6. Biomarkers and Age at 1st Birth

	log(Fibrinogen)		log(C-Reactive Protein)		Glycated Haemoglobin	
	**Men**	**Women**	**Men**	**Women**	**Men**	**Women**
**Age at 1st Birth, Ref: 23-27 (M)/20-24 (W)**	*B/CI*	*B/CI*	*B/CI*	*B/CI*	*B/CI*	*B/CI*
<23(M)-<20(W)	0.005	0.013	0.157**	0.100	-0.015	0.018
	-0.02,0.03	-0.01,0.04	0.03,0.28	-0.05,0.25	-0.10,0.07	-0.05,0.09
28-32 (M) / 25-29 (W)	-0.004	-0.006	-0.010	-0.118**	-0.069**	-0.017
	-0.02,0.01	-0.03,0.01	-0.11,0.09	-0.23,-0.00	-0.14,-0.00	-0.07,0.04
33-38 (M) / 30-34 (W)	-0.013	-0.027**	0.074	-0.135*	-0.081**	-0.030
	-0.03,0.01	-0.05,-0.00	-0.05,0.20	-0.28,0.01	-0.16,-0.00	-0.10,0.04
39+ (M) / 35+ (W)	-0.007	-0.037**	0.001	-0.155	-0.125*	0.008
	-0.04,0.03	-0.07,-0.01	-0.20,0.20	-0.35,0.04	-0.26,0.01	-0.08,0.10
Age at Interview	-0.013	-0.018*	-0.059	-0.016	-0.134***	-0.106***
	-0.03,0.01	-0.04,0.00	-0.16,0.05	-0.13,0.10	-0.20,-0.06	-0.16,-0.05
Education (Ref: Low)						
Medium	-0.012	-0.026**	-0.113*	-0.170***	-0.023	-0.059*
	-0.03,0.01	-0.05,-0.01	-0.23,0.01	-0.30,-0.04	-0.10,0.06	-0.12,0.00
High	-0.029**	-0.020	-0.232***	-0.245***	-0.005	-0.051
	-0.06,-0.00	-0.05,0.01	-0.39,-0.07	-0.42,-0.07	-0.11,0.10	-0.13,0.03
Number of Partnerships (Ref: 0)						
1	-0.056	-0.092**	0.312	0.001	-0.012	-0.031
	-0.21,0.10	-0.18,-0.00	-0.57,1.19	-0.53,0.53	-0.60,0.58	-0.29,0.23
2+	-0.049	-0.110**	0.378	-0.031	0.018	-0.058
	-0.20,0.11	-0.20,-0.02	-0.50,1.26	-0.56,0.50	-0.57,0.61	-0.32,0.20
Ever Unemployed (1978-2001)	0.003	0.004	-0.035	-0.029	0.066**	0.014
	-0.01,0.02	-0.01,0.02	-0.12,0.05	-0.13,0.08	0.01,0.12	-0.04,0.06
Social Class at Birth - Manual	-0.001	0.011	0.030	0.135**	-0.101***	0.044
	-0.02,0.02	-0.01,0.03	-0.08,0.14	0.02,0.25	-0.18,-0.03	-0.01,0.10
Ethnicity (White vs Non-White)	-0.015	0.017	-0.240*	0.093	-0.361***	-0.151*
	-0.06,0.03	-0.04,0.07	-0.51,0.03	-0.25,0.43	-0.54,-0.18	-0.31,0.01
Special Educ. Treatment - Age 11	-0.059*	-0.023	-0.301	-0.556**	0.066	-0.478**
	-0.12,0.01	-0.11,0.06	-0.69,0.09	-1.08,-0.03	-0.17,0.31	-0.92,-0.03
Financial Hardship - Age 11	0.007	0.025*	-0.034	0.085	-0.053	0.026
	-0.02,0.04	-0.00,0.05	-0.20,0.13	-0.08,0.25	-0.17,0.06	-0.05,0.11
Overcrowding - Age 11	-0.002	-0.005	0.053	-0.003	0.019	0.002
	-0.03,0.02	-0.03,0.02	-0.10,0.20	-0.16,0.16	-0.08,0.12	-0.08,0.08
Housing Index (No access to…) - Age 11	0.004	0.005	0.073	0.006	0.108**	0.036
	-0.02,0.03	-0.02,0.03	-0.08,0.22	-0.16,0.17	0.00,0.21	-0.04,0.11
Birth Weight	0.000	0.000	-0.001	-0.001	0.000	0.000
	-0.00,0.00	-0.00,0.00	-0.00,0.00	-0.00,0.00	-0.00,0.00	-0.00,0.00
Mother smoked during pregnancy	0.009	-0.015*	0.078*	-0.091*	0.049	0.015

	-0.01,0.02	-0.03,0.00	-0.01,0.17	-0.19,0.01	-0.01,0.11	-0.03,0.06
BSGA Score - Age 11	0.000	0.000	-0.002	-0.003	0.003*	0.002
	-0.00,0.00	-0.00,0.00	-0.01,0.00	-0.01,0.00	-0.00,0.01	-0.00,0.01
Parents interested in Edu - Age 11	-0.009	-0.019*	-0.076	-0.099	-0.053	0.007
	-0.03,0.01	-0.04,0.00	-0.19,0.04	-0.23,0.03	-0.13,0.02	-0.06,0.07
Teenage Smoking - Age 16	0.039***	0.017*	0.242***	0.085	0.049	0.049*
	0.02,0.06	-0.00,0.03	0.15,0.34	-0.03,0.20	-0.01,0.11	-0.00,0.10
Rutter - Age 7	0.000	0.002	-0.026	0.017	-0.026	-0.010
	-0.01,0.01	-0.01,0.01	-0.07,0.02	-0.04,0.07	-0.06,0.01	-0.04,0.01
Rutter - Age 11	0.000	0.008*	0.020	0.052*	0.017	0.017
	-0.01,0.01	-0.00,0.02	-0.03,0.07	-0.00,0.11	-0.01,0.05	-0.01,0.04
Parents' Years of Educ.	-0.006*	-0.006*	-0.023	-0.008	-0.011	-0.002
	-0.01,0.00	-0.01,0.00	-0.06,0.01	-0.05,0.03	-0.03,0.01	-0.02,0.01
Mother stayd in school after min. age	0.010	-0.021*	-0.014	-0.160**	-0.015	-0.005
	-0.01,0.03	-0.04,0.00	-0.13,0.10	-0.30,-0.02	-0.09,0.06	-0.07,0.06
Family Difficulties - Age 7	0.002	0.033	0.266*	-0.032	0.289**	-0.113
	-0.06,0.06	-0.02,0.08	-0.05,0.58	-0.33,0.27	0.07,0.51	-0.25,0.03
Parents Divorced by age 10/11	-0.029	-0.033	-0.125	0.048	-0.157	0.093
	-0.08,0.02	-0.08,0.01	-0.43,0.18	-0.23,0.32	-0.36,0.04	-0.04,0.22
Times hospitalized - Age 11	0.005	0.006	0.022	0.036	0.006	-0.018
	-0.00,0.01	-0.00,0.02	-0.02,0.07	-0.02,0.09	-0.03,0.04	-0.05,0.01
Out of school for 1+ months - Age 11	0.024	0.020	0.088	0.045	-0.046	0.042
	-0.01,0.06	-0.02,0.06	-0.12,0.29	-0.18,0.27	-0.18,0.09	-0.06,0.14
General Ability - Age 11	-0.001*	0.000	-0.002	-0.003	-0.003**	0.000
	-0.00,0.00	-0.00,0.00	-0.01,0.00	-0.01,0.00	-0.01,-0.00	-0.00,0.00
Enuresis - Age 7	0.013	-0.005	0.073	-0.076	0.004	-0.018
	-0.01,0.04	-0.03,0.02	-0.07,0.22	-0.24,0.09	-0.09,0.10	-0.09,0.06
Enuresis - Age 11	-0.003	0.022	-0.036	0.056	-0.025	-0.020
	-0.04,0.03	-0.02,0.06	-0.23,0.16	-0.19,0.30	-0.16,0.11	-0.13,0.09
Physical Coordination Problems - Age 11	0.009	0.047***	0.061	0.288***	-0.009	0.101**
	-0.01,0.03	0.02,0.07	-0.07,0.20	0.13,0.45	-0.10,0.08	0.02,0.18
Constant	1.881***	2.151***	3.270	1.818	12.000***	10.679***
	1.03,2.73	1.28,3.02	-1.54,8.08	-3.50,7.13	8.81,15.19	8.10,13.25
N	2,823	3,035	2,833	3,034	2,908	3,118

* p<0.10, ** p<0.05, *** p<0.01

Table A6 Cont. Biomarkers and Age at 1st Birth

	**Cholesterol Ratio (Tot./HDL)**		**High Blood Pressure**		**Obesity**	
	**Men**	**Women**	**Men**	**Women**	**Men**	**Women**
**Age at 1st Birth, Ref: 23-27 (M)/20-24 (W)**	*B/CI*	*B/CI*	*OR/CI*	*OR/CI*	*OR/CI*	*OR/CI*
<23(M)-<20(W)	0.059	0.075	0.978	1.009	1.310**	1.003
	-0.07,0.19	-0.04,0.19	0.74,1.30	0.63,1.61	1.04,1.65	0.79,1.28

28-32 (M) / 25-29 (W)	-0.034	-0.088*	1.060	1.136	1.017	0.725***
	-0.15,0.08	-0.18,0.00	0.83,1.35	0.79,1.63	0.83,1.25	0.59,0.89
33-38 (M) / 30-34 (W)	-0.069	-0.217***	0.810	0.520**	0.770**	0.592***
	-0.20,0.06	-0.33,-0.11	0.60,1.10	0.30,0.91	0.59,1.00	0.45,0.77
39+ (M) / 35+ (W)	0.019	-0.152**	0.801	0.798	0.818	0.671**
	-0.20,0.24	-0.30,-0.00	0.48,1.33	0.40,1.57	0.53,1.26	0.47,0.97
Age at Interview	-0.017	-0.009	0.843	1.204	0.951	1.079
	-0.13,0.10	-0.10,0.08	0.66,1.08	0.83,1.76	0.77,1.17	0.88,1.32
Education (Ref: Low)						
Medium	-0.031	-0.151***	1.080	0.921	1.111	0.900
	-0.17,0.10	-0.25,-0.05	0.81,1.43	0.61,1.38	0.87,1.41	0.72,1.12
High	-0.083	-0.113*	1.084	0.957	0.909	0.866
	-0.26,0.10	-0.24,0.02	0.73,1.60	0.54,1.69	0.65,1.27	0.64,1.17
Number of Partnerships (Ref: 0)						
1	0.422	0.114	0.451	0.739	2.391	0.481*
	-0.54,1.38	-0.31,0.54	0.09,2.34	0.16,3.32	0.26,21.85	0.22,1.05
2+	0.386	0.093	0.483	0.651	2.315	0.415**
	-0.57,1.35	-0.33,0.51	0.09,2.50	0.15,2.92	0.25,21.13	0.19,0.90
Ever Unemployed (1978-2001)	0.054	0.076*	0.932	0.893	0.973	1.186*
	-0.04,0.15	-0.00,0.16	0.76,1.15	0.63,1.26	0.82,1.16	0.99,1.42
Social Class at Birth - Manual	0.080	0.137***	1.235	1.590**	1.109	1.212*
	-0.04,0.20	0.04,0.23	0.96,1.59	1.04,2.43	0.89,1.38	0.98,1.50
Ethnicity (White vs Non-White)	-0.064	0.246*	0.734	1.763	1.352	0.909
	-0.35,0.22	-0.01,0.50	0.42,1.30	0.52,5.96	0.77,2.38	0.52,1.58
Special Educ. Treatment - Age 11	-0.227	-0.390*	1.388	0.679	0.761	0.672
	-0.61,0.15	-0.84,0.06	0.57,3.37	0.19,2.44	0.39,1.48	0.32,1.39
Financial Hardship - Age 11	-0.070	0.085	1.202	0.642	0.996	1.043
	-0.24,0.10	-0.05,0.21	0.84,1.71	0.36,1.15	0.73,1.36	0.79,1.38
Overcrowding - Age 11	0.010	-0.023	0.964	1.141	1.045	0.769*
	-0.15,0.17	-0.15,0.10	0.69,1.34	0.69,1.88	0.79,1.38	0.58,1.02
Housing Index (No access to…) - Age 11	-0.314***	0.044	1.200	0.725	0.782	1.125
	-0.47,-0.15	-0.08,0.17	0.86,1.67	0.41,1.27	0.57,1.07	0.86,1.47
Birth Weight	0.000	-0.002**	0.995**	0.983***	1.004**	1.003
	-0.00,0.00	-0.00,-0.00	0.99,1.00	0.97,0.99	1.00,1.01	1.00,1.01
Mother smoked during pregnancy	-0.045	0.027	0.966	0.926	1.284***	1.188*
	-0.14,0.05	-0.05,0.11	0.78,1.20	0.67,1.28	1.08,1.53	1.00,1.41
BSGA Score - Age 11	0.002	0.000	0.993	0.997	1.004	1.003
	-0.00,0.01	-0.01,0.01	0.98,1.01	0.97,1.02	0.99,1.01	0.99,1.02
Parents interested in Edu - Age 11	-0.010	-0.043	1.066	1.091	0.907	0.896
	-0.13,0.11	-0.14,0.05	0.82,1.39	0.73,1.63	0.72,1.14	0.72,1.11
Teenage Smoking - Age 16	0.152***	0.130***	1.006	0.916	0.998	0.759***
	0.05,0.26	0.04,0.22	0.79,1.28	0.63,1.32	0.81,1.22	0.62,0.93
Rutter - Age 7	-0.039	-0.004	0.969	1.126	0.927	1.036

	-0.09,0.01	-0.04,0.04	0.87,1.09	0.95,1.34	0.84,1.02	0.95,1.14
Rutter - Age 11	0.072***	0.047**	1.044	1.013	1.043	1.130**
	0.02,0.12	0.01,0.09	0.93,1.17	0.85,1.21	0.95,1.15	1.03,1.24
Parents' Years of Educ.	-0.028*	-0.024	0.932	1.060	0.937*	0.969
	-0.06,0.00	-0.05,0.01	0.86,1.01	0.94,1.19	0.87,1.01	0.90,1.04
Mother stayd in school after min. age	0.054	-0.079	1.010	0.950	0.845	0.909
	-0.07,0.18	-0.18,0.03	0.76,1.33	0.61,1.49	0.66,1.08	0.71,1.17
Family Difficulties - Age 7	-0.085	0.093	1.343	0.453	1.594	1.053
	-0.42,0.25	-0.14,0.33	0.68,2.64	0.14,1.48	0.90,2.83	0.63,1.76
Parents Divorced by age 10/11	0.241	-0.140	0.720	1.570	0.895	1.050
	-0.09,0.57	-0.36,0.07	0.36,1.46	0.67,3.67	0.50,1.60	0.66,1.68
Times hospitalized - Age 11	-0.026	0.000	0.909	0.865	1.002	1.078
	-0.08,0.02	-0.04,0.04	0.81,1.02	0.71,1.06	0.91,1.10	0.98,1.19
Out of school for 1+ months - Age 11	0.189*	0.023	1.318	0.986	1.045	1.120
	-0.03,0.41	-0.15,0.19	0.85,2.04	0.48,2.01	0.70,1.55	0.79,1.59
General Ability - Age 11	0.000	0.000	0.999	1.001	0.998	0.997
	-0.00,0.00	-0.00,0.00	0.99,1.01	0.99,1.01	0.99,1.01	0.99,1.00
Enuresis - Age 7	-0.064	-0.012	0.847	1.243	0.861	0.968
	-0.22,0.09	-0.14,0.11	0.60,1.19	0.78,1.98	0.65,1.15	0.74,1.27
Enuresis - Age 11	0.135	0.030	1.014	1.389	1.144	1.100
	-0.07,0.34	-0.15,0.21	0.65,1.59	0.71,2.71	0.79,1.66	0.73,1.66
Physical Coordination Problems - Age 11	0.169**	0.208***	1.061	1.280	1.048	1.385**
	0.02,0.31	0.08,0.33	0.78,1.44	0.80,2.04	0.80,1.37	1.07,1.79
Constant	5.371**	4.543**	3491.980	0.000	1.526	0.047
	0.15,10.59	0.43,8.65	0.04,2.80e +08	0.00,1778.12	0.00,26441.73	0.00,544.96
N	2,873	3,072	3,310	3,632	3,299	3,629

* p<0.10, ** p<0.05, *** p<0.01

Table A6 Cont. Biomarkers and Age at 1st Birth

	**Waist-Hip Ratio*10**		**FEV1/FVC**		**Metabolic Syndrome**	
	**Men**	**Women**	**Men**	**Women**	**Men**	**Women**
**Age at 1st Birth, Ref: 23-27 (M)/20-24 (W)**	*B/CI*	*B/CI*	*B/CI*	*B/CI*	*OR/CI*	*OR/CI*
<23(M)-<20(W)	0.091***	0.036	-0.004	-0.016**	1.216*	1.279*
	0.03,0.15	-0.03,0.10	-0.02,0.01	-0.03,-0.00	0.97,1.52	1.00,1.64
28-32 (M) / 25-29 (W)	0.013	-0.036	-0.006	0.001	1.119	0.879
	-0.04,0.06	-0.09,0.02	-0.02,0.00	-0.01,0.01	0.93,1.35	0.71,1.08
33-38 (M) / 30-34 (W)	-0.040	-0.039	-0.009	-0.005	0.776**	0.798*
	-0.10,0.02	-0.10,0.02	-0.02,0.00	-0.02,0.01	0.62,0.97	0.61,1.04
39+ (M) / 35+ (W)	-0.055	-0.100**	0.006	-0.016*	0.766	0.694*
	-0.15,0.04	-0.19,-0.01	-0.01,0.03	-0.03,0.00	0.53,1.12	0.48,1.01
Age at Interview	-0.029	0.079***	0.000	-0.007	0.806**	0.935
	-0.08,0.02	0.03,0.13	-0.01,0.01	-0.02,0.00	0.67,0.98	0.76,1.15
Education (Ref: Low)						

Medium	-0.070**	-0.063**	0.009	0.004	0.890	0.963
	-0.13,-0.01	-0.12,-0.01	-0.00,0.02	-0.01,0.02	0.71,1.11	0.77,1.20
High	-0.114***	-0.048	0.014*	-0.007	0.901	0.948
	-0.20,-0.03	-0.13,0.03	-0.00,0.03	-0.02,0.01	0.67,1.22	0.70,1.29
Number of Partnerships (Ref: 0)						
1	-0.314	-0.125	-0.037	0.015	1.278	0.770
	-0.74,0.12	-0.35,0.10	-0.13,0.06	-0.03,0.06	0.20,8.04	0.30,1.96
2+	-0.322	-0.117	-0.036	0.011	1.323	0.700
	-0.75,0.11	-0.34,0.10	-0.13,0.06	-0.03,0.06	0.21,8.32	0.28,1.78
Ever Unemployed (1978-2001)	-0.004	0.024	-0.002	0.003	1.015	1.145
	-0.05,0.04	-0.02,0.07	-0.01,0.01	-0.01,0.01	0.86,1.19	0.95,1.38
Social Class at Birth - Manual	0.025	0.061**	0.005	0.003	1.053	1.490***
	-0.03,0.08	0.01,0.11	-0.01,0.02	-0.01,0.01	0.87,1.28	1.20,1.86
Ethnicity (White vs Non-White)	0.021	0.065	-0.018	0.000	1.121	1.333
	-0.11,0.15	-0.08,0.21	-0.05,0.01	-0.03,0.03	0.68,1.85	0.73,2.44
Special Educ. Treatment - Age 11	-0.031	-0.361***	-0.005	-0.027	0.995	0.624
	-0.21,0.15	-0.58,-0.14	-0.04,0.03	-0.07,0.02	0.51,1.93	0.27,1.42
Financial Hardship - Age 11	-0.082**	0.044	0.002	-0.008	0.722**	0.909
	-0.16,-0.00	-0.03,0.12	-0.02,0.02	-0.02,0.01	0.54,0.96	0.68,1.21
Overcrowding - Age 11	0.011	0.019	-0.006	0.005	0.811	0.998
	-0.06,0.08	-0.06,0.09	-0.02,0.01	-0.01,0.02	0.62,1.06	0.76,1.31
Housing Index (No access to…) - Age 11	-0.012	0.044	-0.009	0.007	0.903	1.024
	-0.09,0.06	-0.03,0.12	-0.03,0.01	-0.01,0.02	0.68,1.19	0.78,1.35
Birth Weight	0.001	0.001	0.000	0.000	1.004*	0.996
	-0.00,0.00	-0.00,0.00	-0.00,0.00	-0.00,0.00	1.00,1.01	0.99,1.00
Mother smoked during pregnancy	0.064***	0.066***	-0.007	-0.007	1.096	1.000
	0.02,0.11	0.02,0.11	-0.02,0.00	-0.02,0.00	0.93,1.29	0.84,1.20
BSGA Score - Age 11	0.003*	0.000	0.000	0.000	1.000	0.986**
	-0.00,0.01	-0.00,0.00	-0.00,0.00	-0.00,0.00	0.99,1.01	0.97,1.00
Parents interested in Edu - Age 11	-0.013	-0.022	0.008	0.000	0.916	0.831
	-0.07,0.04	-0.08,0.04	-0.00,0.02	-0.01,0.01	0.74,1.13	0.67,1.04
Teenage Smoking - Age 16	0.058**	0.067***	-0.015***	-0.010**	1.233**	1.084
	0.01,0.11	0.02,0.12	-0.03,-0.00	-0.02,-0.00	1.03,1.48	0.89,1.32
Rutter - Age 7	-0.016	0.006	-0.001	0.001	0.871***	1.071
	-0.04,0.01	-0.02,0.03	-0.01,0.00	-0.00,0.01	0.80,0.95	0.97,1.18
Rutter - Age 11	0.013	0.019	-0.004	-0.002	1.075	0.990
	-0.01,0.04	-0.01,0.04	-0.01,0.00	-0.01,0.00	0.98,1.17	0.90,1.09
Parents' Years of Educ.	-0.022***	-0.009	-0.003*	0.001	0.958	0.962
	-0.04,-0.01	-0.03,0.01	-0.01,0.00	-0.00,0.00	0.90,1.02	0.89,1.04
Mother stayd in school after min. age	-0.024	0.030	0.003	-0.001	0.979	0.923
	-0.08,0.03	-0.03,0.09	-0.01,0.02	-0.01,0.01	0.79,1.21	0.71,1.19

Family Difficulties - Age 7	0.025	0.005	0.010	0.018	1.350	1.099
	-0.13,0.18	-0.13,0.15	-0.02,0.04	-0.01,0.05	0.77,2.37	0.65,1.86
Parents Divorced by age 10/11	-0.093	-0.016	-0.016	-0.029**	0.848	1.040
	-0.24,0.05	-0.14,0.11	-0.05,0.02	-0.05,-0.00	0.49,1.45	0.65,1.67
Times hospitalized - Age 11	-0.004	0.009	0.000	-0.005**	0.934	1.046
	-0.03,0.02	-0.02,0.03	-0.01,0.00	-0.01,-0.00	0.86,1.02	0.95,1.15
Out of school for 1+ months - Age 11	0.067	0.026	-0.008	0.013	0.817	1.251
	-0.03,0.17	-0.07,0.12	-0.03,0.01	-0.01,0.03	0.56,1.19	0.87,1.81
General Ability - Age 11	-0.003***	-0.002**	0.000	0.000	0.996	0.995
	-0.00,-0.00	-0.00,-0.00	-0.00,0.00	-0.00,0.00	0.99,1.00	0.99,1.00
Enuresis - Age 7	-0.045	0.015	-0.004	0.019***	0.923	1.119
	-0.11,0.02	-0.06,0.09	-0.02,0.01	0.01,0.03	0.71,1.20	0.85,1.48
Enuresis - Age 11	0.012	0.034	0.011	-0.025**	1.038	0.895
	-0.08,0.10	-0.08,0.14	-0.01,0.03	-0.05,-0.00	0.74,1.46	0.59,1.36
Physical Coordination Problems - Age 11	0.112***	0.149***	-0.007	0.008	1.234*	1.759***
	0.05,0.18	0.08,0.22	-0.02,0.01	-0.01,0.02	0.96,1.58	1.35,2.29
Constant	11.208***	5.030***	0.893***	1.080***	13745.187**	33.689
	8.87,13.55	2.67,7.39	0.40,1.39	0.60,1.56	1.98,9.55e+07	0.00,458026.66
N	3,325	3,665	3,255	3,588	3,007	3,260

* p<0.10, ** p<0.05, *** p<0.01

Figure A5. Age at Last Birth and Biomarkers (No controls)

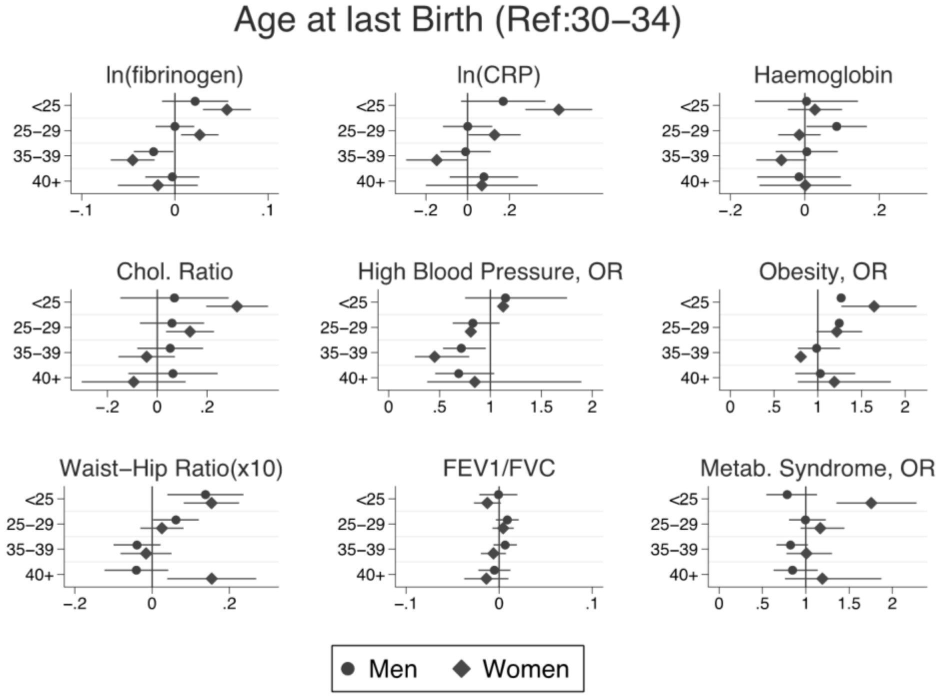

Figure A6. Age at Last Birth and Biomarkers (Restricted set of confounders: father's social class at birth, parents divorced at age 11, overcrowding at age 11, housing conditions at age 11, teenage smoking, number of times hospitalized at age 11, out of school for more than a month at age 11, parents' years of education, education level, number of partnerships, ever unemployed between 1978 and 2002**)**

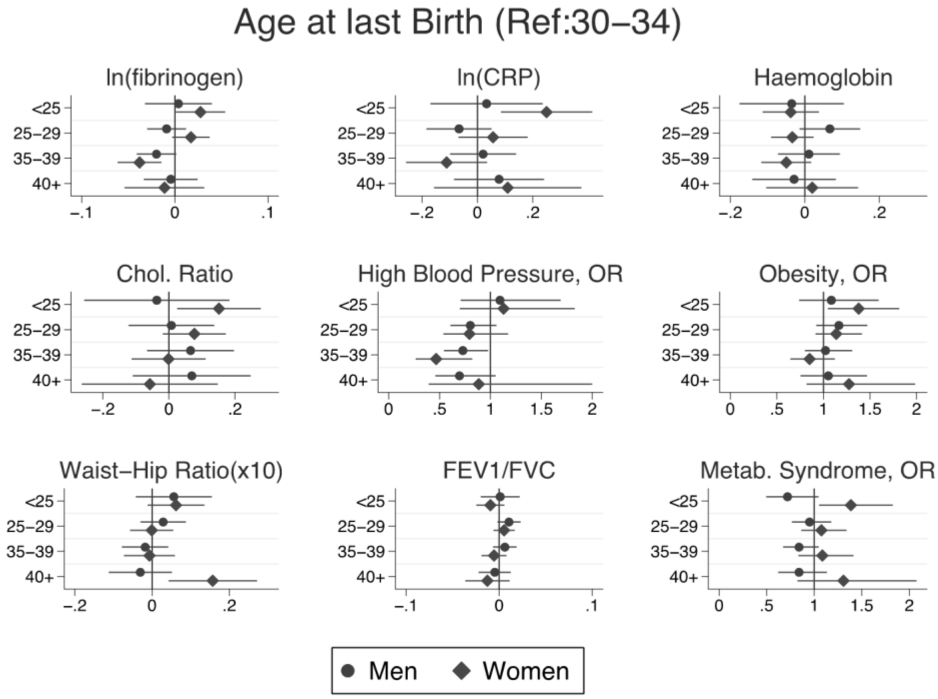

Table A7. Biomarkers and Age at last Birth

	log(Fibrinogen)		log(C-Reactive Protein)		Glycated Haemoglobin	
	**Men**	**Women**	**Men**	**Women**	**Men**	**Women**
**Age at Last Birth, Ref: 30-34**	*B/CI*	*B/CI*	*B/CI*	*B/CI*	*B/CI*	*B/CI*
<25	-0.001	0.026*	0.002	0.239***	-0.051	-0.041
	-0.04,0.04	-0.00,0.05	-0.20,0.21	0.07,0.40	-0.19,0.09	-0.12,0.03
25-29	-0.010	0.016	-0.081	0.047	0.062	-0.035
	-0.03,0.01	-0.00,0.04	-0.20,0.04	-0.08,0.17	-0.02,0.14	-0.09,0.02
35-39	-0.021*	-0.037***	0.004	-0.112	-0.006	-0.055
	-0.04,0.00	-0.06,-0.01	-0.12,0.12	-0.26,0.03	-0.09,0.08	-0.12,0.01
40+	-0.005	-0.012	0.054	0.104	-0.038	0.010
	-0.03,0.02	-0.05,0.03	-0.11,0.22	-0.16,0.37	-0.15,0.07	-0.11,0.13
Age at Interview	-0.012	-0.020*	-0.036	-0.031	-0.145***	-0.089***
	-0.03,0.01	-0.04,0.00	-0.15,0.08	-0.16,0.10	-0.23,-0.07	-0.15,-0.03
Education (Ref: Low)						
Medium	-0.004	-0.031***	-0.073	-0.183***	-0.027	-0.091***
	-0.03,0.02	-0.05,-0.01	-0.21,0.06	-0.32,-0.05	-0.12,0.07	-0.15,-0.03
High	-0.020	-0.021	-0.194**	-0.251***	-0.018	-0.076*
	-0.05,0.01	-0.05,0.01	-0.37,-0.02	-0.44,-0.06	-0.14,0.10	-0.16,0.01
Number of Partnerships (Ref: 0)						
1	0.207	-0.103	0.24	-0.2	-0.08	-0.019
	-0.18,0.59	-0.27,0.06	-1.94,2.42	-1.21,0.81	-1.58,1.42	-0.49,0.45
2+	0.215	-0.111	0.274	-0.199	-0.025	-0.045
	-0.17,0.60	-0.27,0.05	-1.90,2.45	-1.21,0.81	-1.53,1.48	-0.52,0.42
Ever Unemployed (1978-2001)	-0.004	0.008	-0.024	-0.026	0.068*	0.02
	-0.02,0.01	-0.01,0.03	-0.12,0.07	-0.14,0.09	-0.00,0.14	-0.03,0.07
Social Class at Birth - Manual	0.001	0.008	0.052	0.142**	-0.101**	0.033
	-0.02,0.02	-0.01,0.03	-0.06,0.17	0.01,0.27	-0.19,-0.02	-0.03,0.09
Ethnicity (White vs Non-White)	0.001	0.02	-0.24	0.262	-0.424***	-0.145
	-0.05,0.05	-0.04,0.08	-0.54,0.06	-0.13,0.65	-0.63,-0.22	-0.32,0.03
Special Educ. Treatment - Age 11	-0.064	-0.037	-0.297	-0.641**	0.085	-0.599**
	-0.14,0.02	-0.13,0.05	-0.78,0.18	-1.23,-0.06	-0.23,0.40	-1.14,-0.06
Financial Hardship - Age 11	0.015	0.016	0.023	0.054	-0.06	0.045
	-0.02,0.05	-0.01,0.05	-0.16,0.20	-0.13,0.24	-0.19,0.07	-0.04,0.13
Overcrowding - Age 11	-0.004	-0.003	0.043	-0.01	0.009	0.005
	-0.03,0.03	-0.03,0.03	-0.12,0.20	-0.19,0.17	-0.11,0.12	-0.08,0.09
Housing Index (No access to…) - Age 11	-0.001	0.007	0.081	0.052	0.131**	0.05
	-0.03,0.03	-0.02,0.04	-0.08,0.25	-0.13,0.23	0.01,0.25	-0.04,0.13
Birth Weight	0	-0.001**	-0.001	-0.001	0	-0.001
	-0.00,0.00	-0.00,-0.00	-0.00,0.00	-0.00,0.00	-0.00,0.00	-0.00,0.00
Mother smoked during pregnancy	0.012	-0.016*	0.108**	-0.062	0.068*	0.012
	-0.01,0.03	-0.03,0.00	0.01,0.21	-0.17,0.05	-0.00,0.14	-0.04,0.06
BSGA Score - Age 11	0.001	-0.001	0	-0.002	0.005**	0.003
	-0.00,0.00	-0.00,0.00	-0.01,0.01	-0.01,0.01	0.00,0.01	-0.00,0.01
Parents interested in Edu - Age 11	-0.012	-0.024**	-0.099	-0.072	-0.038	-0.006
	-0.04,0.01	-0.05,-0.00	-0.23,0.03	-0.22,0.07	-0.13,0.05	-0.07,0.06

Teenage Smoking - Age 16	0.037***	0.017*	0.252***	0.076	0.061*	0.050*
	0.02,0.06	-0.00,0.04	0.15,0.36	-0.05,0.20	-0.01,0.13	-0.00,0.10
Rutter - Age 7	-0.001	0	-0.016	0.001	-0.016	-0.01
	-0.01,0.01	-0.01,0.01	-0.07,0.04	-0.06,0.06	-0.05,0.02	-0.04,0.02
Rutter - Age 11	-0.002	0.011**	0.01	0.062**	0.007	0.013
	-0.01,0.01	0.00,0.02	-0.04,0.06	0.00,0.12	-0.03,0.04	-0.01,0.04
Parents' Years of Educ.	-0.005	-0.007**	-0.024	-0.008	-0.011	0.001
	-0.01,0.00	-0.01,-0.00	-0.06,0.01	-0.05,0.03	-0.03,0.01	-0.02,0.02
Mother stayed in school after min. age	0.004	-0.017	-0.029	-0.159**	-0.027	-0.015
	-0.02,0.03	-0.04,0.01	-0.16,0.10	-0.31,-0.01	-0.12,0.06	-0.08,0.05
Family Difficulties - Age 7	-0.004	0.032	0.229	-0.052	0.205	-0.108
	-0.07,0.06	-0.02,0.09	-0.13,0.58	-0.38,0.27	-0.07,0.48	-0.26,0.04
Parents Divorced by age 10/11	-0.001	-0.029	-0.098	0.073	-0.049	0.086
	-0.07,0.06	-0.08,0.02	-0.45,0.26	-0.23,0.37	-0.30,0.20	-0.05,0.22
Times hospitalized - Age 11	0.006	0.004	0.025	0.024	0.005	-0.026*
	-0.00,0.02	-0.01,0.01	-0.03,0.08	-0.04,0.09	-0.03,0.04	-0.05,0.00
Out of school for 1+ months - Age 11	0.011	0.022	0.156	0.079	-0.092	0.055
	-0.03,0.05	-0.02,0.06	-0.08,0.39	-0.17,0.33	-0.25,0.07	-0.05,0.16
General Ability - Age 11	0	-0.001	-0.002	-0.004*	-0.003**	0
	-0.00,0.00	-0.00,0.00	-0.01,0.00	-0.01,0.00	-0.01,-0.00	-0.00,0.00
Enuresis - Age 7	0.004	-0.002	0.027	-0.047	-0.04	-0.002
	-0.02,0.03	-0.03,0.03	-0.13,0.19	-0.23,0.13	-0.15,0.07	-0.08,0.08
Enuresis - Age 11	0.004	0.012	0.075	-0.011	0.006	-0.028
	-0.04,0.04	-0.03,0.05	-0.14,0.29	-0.28,0.26	-0.15,0.17	-0.15,0.09
Physical Coordination Problems - Age 11	0.004	0.050***	0.062	0.294***	-0.022	0.098**
	-0.02,0.03	0.02,0.08	-0.09,0.22	0.12,0.47	-0.12,0.08	0.02,0.18
Constant	1.540***	2.298***	2.245	2.544	12.592***	10.060***
	0.52,2.57	1.34,3.25	-3.51,8.00	-3.41,8.50	8.62,16.57	7.28,12.84
N	2,197	2,501	2,205	2,500	2,259	2,565

* p<0.10, ** p<0.05, *** p<0.01

Table A7 Cont. Biomarkers and Age at last Birth

	**Cholesterol Ratio (Tot./HDL)**		**High Blood Pressure**		**Obesity**	
	**Men**	**Women**	**Men**	**Women**	**Men**	**Women**
**Age at Last Birth, Ref: 30-34**	*B/CI*	*B/CI*	*OR/CI*	*OR/CI*	*OR/CI*	*OR/CI*
<25	-0.019	0.136**	1.101	1.087	1.051	1.334**
	-0.24,0.20	0.01,0.26	0.71,1.71	0.67,1.78	0.71,1.55	1.01,1.77
25-29	0.022	0.069	0.819	0.773	1.134	1.145
	-0.11,0.15	-0.03,0.16	0.62,1.09	0.52,1.15	0.90,1.44	0.92,1.43
35-39	0.069	-0.003	0.729**	0.467***	0.995	0.849
	-0.06,0.20	-0.12,0.11	0.54,0.98	0.26,0.83	0.78,1.28	0.64,1.12
40+	0.067	-0.061	0.713	0.847	1.038	1.295
	-0.11,0.25	-0.27,0.14	0.47,1.08	0.37,1.94	0.74,1.46	0.83,2.03
Age at Interview	-0.016	-0.003	0.775*	1.166	0.956	1.161
	-0.14,0.11	-0.10,0.10	0.59,1.03	0.77,1.77	0.76,1.21	0.93,1.46
Education (Ref: Low)						
Medium	-0.045	-0.176***	1.195	0.957	1.138	0.805*

	-0.20,0.11	-0.28,-0.07	0.87,1.65	0.60,1.51	0.87,1.49	0.64,1.02
High	-0.126	-0.166**	1.173	0.979	0.864	0.811
	-0.33,0.08	-0.31,-0.02	0.76,1.82	0.52,1.84	0.59,1.26	0.58,1.13
Number of Partnerships (Ref: 0)						
1	1.200	0.035	0.944	1.097	1.069	0.162**
	-1.26,3.66	-0.82,0.89	0.75,1.18	0.79,1.53	0.89,1.29	0.04,0.74
2+	1.129	0.032	1.000	1.000	1.000	0.148**
	-1.33,3.58	-0.82,0.89				0.03,0.67
Ever Unemployed (1978-2001)	0.037	0.028	0.875	0.811	0.972	1.108
	-0.07,0.15	-0.06,0.12	0.69,1.12	0.54,1.21	0.80,1.19	0.90,1.36
Social Class at Birth - Manual	0.124*	0.140***	1.241	1.852**	1.151	1.219
	-0.01,0.26	0.04,0.24	0.93,1.66	1.15,2.99	0.90,1.47	0.96,1.55
Ethnicity (White vs Non-White)	-0.008	0.209	0.660	1.502	1.313	0.927
	-0.32,0.31	-0.07,0.49	0.35,1.23	0.43,5.20	0.71,2.42	0.49,1.74
Special Educ. Treatment - Age 11	-0.460*	-0.488*	1.103	0.495	0.663	0.615
	-0.93,0.01	-0.99,0.01	0.41,3.00	0.13,1.84	0.29,1.50	0.28,1.36
Financial Hardship - Age 11	0.002	0.109	1.354	0.624	1.003	1.109
	-0.19,0.20	-0.03,0.25	0.92,1.99	0.32,1.21	0.71,1.41	0.82,1.50
Overcrowding - Age 11	0.009	-0.026	0.858	0.988	1.051	0.705**
	-0.17,0.18	-0.16,0.11	0.58,1.26	0.56,1.76	0.77,1.44	0.51,0.97
Housing Index (No access to…) - Age 11	-0.306***	0.093	1.175	0.539*	0.842	1.213
	-0.48,-0.13	-0.04,0.23	0.81,1.72	0.27,1.08	0.60,1.19	0.91,1.62
Birth Weight	-0.001	-0.002	0.995*	0.984***	1.004	1.003
	-0.00,0.00	-0.00,0.00	0.99,1.00	0.98,0.99	1.00,1.01	1.00,1.01
Mother smoked during pregnancy	-0.049	0.032	1.003	0.991	1.226**	1.228**
	-0.16,0.06	-0.05,0.12	0.79,1.28	0.69,1.42	1.00,1.50	1.01,1.49
BSGA Score - Age 11	0.004	-0.001	0.994	0.997	1.007	1.001
	-0.00,0.01	-0.01,0.00	0.98,1.01	0.97,1.02	0.99,1.02	0.99,1.01
Parents interested in Edu - Age 11	-0.008	-0.053	1.133	1.160	0.906	0.909
	-0.15,0.13	-0.16,0.06	0.83,1.55	0.73,1.83	0.70,1.17	0.71,1.16
Teenage Smoking - Age 16	0.132**	0.161***	1.051	0.919	1.002	0.791**
	0.01,0.25	0.07,0.26	0.80,1.38	0.61,1.38	0.80,1.25	0.64,0.99
Rutter - Age 7	-0.031	-0.008	0.969	1.127	0.982	1.029
	-0.09,0.03	-0.05,0.04	0.85,1.10	0.93,1.36	0.88,1.09	0.93,1.14
Rutter - Age 11	0.080***	0.048**	1.027	0.980	1.025	1.137**
	0.02,0.14	0.00,0.09	0.90,1.17	0.80,1.20	0.92,1.14	1.02,1.26
Parents' Years of Educ.	-0.035*	-0.030*	0.926	1.108	0.930*	0.942
	-0.07,0.00	-0.06,0.00	0.84,1.02	0.98,1.26	0.86,1.01	0.87,1.02
Mother stayed in school after min. age	0.067	-0.102*	1.120	0.851	0.898	0.868
	-0.07,0.21	-0.22,0.01	0.82,1.53	0.52,1.40	0.69,1.17	0.66,1.15
Family Difficulties - Age 7	-0.071	0.110	1.312	0.515	1.789*	1.215
	-0.47,0.33	-0.15,0.37	0.61,2.81	0.13,1.96	0.92,3.46	0.70,2.12
Parents Divorced by age 10/11	0.188	-0.155	0.941	1.195	0.949	0.943
	-0.21,0.59	-0.39,0.08	0.44,2.03	0.44,3.25	0.48,1.87	0.56,1.59
Times hospitalized - Age 11	-0.034	-0.004	0.904	0.872	1.010	1.052
	-0.09,0.02	-0.05,0.04	0.79,1.03	0.70,1.09	0.91,1.12	0.95,1.17
Out of school for 1+ months - Age 11	0.169	0.010	1.294	1.047	1.083	1.184
	-0.08,0.42	-0.18,0.20	0.78,2.14	0.49,2.25	0.69,1.69	0.81,1.74
General Ability - Age 11	0.000	-0.001	0.997	1.001	0.998	0.995

	-0.00,0.00	-0.00,0.00	0.99,1.01	0.99,1.02	0.99,1.01	0.99,1.00
Enuresis - Age 7	-0.109	-0.020	0.725	1.372	0.848	1.007
	-0.28,0.06	-0.15,0.11	0.48,1.08	0.83,2.26	0.62,1.17	0.75,1.36
Enuresis - Age 11	0.123	-0.006	1.072	1.353	1.101	1.184
	-0.11,0.36	-0.20,0.19	0.65,1.78	0.65,2.81	0.73,1.67	0.76,1.85
Physical Coordination Problems - Age 11	0.188**	0.229***	1.095	1.192	1.018	1.343**
	0.02,0.35	0.09,0.36	0.77,1.55	0.71,2.01	0.75,1.38	1.01,1.79
Constant	4.816	4.463*	1.15e+05*	0.000	3.566	0.006
	-1.55,11.18	-0.12,9.05	0.35,3.78e+10	0.00,18544.15	0.00,153086.46	0.00,228.16
N	2,240	2,530	2,555	2,960	2,556	2,973

* p<0.10, ** p<0.05, *** p<0.01

Table A7 Cont. Biomarkers and Age at last Birth

	Waist-Hip Ratio*10		FEV1/FVC		Metabolic Syndrome	
	**Men**	**Women**	**Men**	**Women**	**Men**	**Women**
**Age at Last Birth, Ref: 30-34**	*B/CI*	*B/CI*	*B/CI*	*B/CI*	*OR/CI*	*OR/CI*
<25	0.045	0.048	0.001	-0.009	0.708*	1.365**
	-0.05,0.14	-0.03,0.12	-0.02,0.02	-0.02,0.01	0.48,1.03	1.03,1.81
25-29	0.022	-0.002	0.011*	0.005	0.941	1.079
	-0.04,0.08	-0.06,0.05	-0.00,0.02	-0.01,0.02	0.76,1.17	0.86,1.35
35-39	-0.028	-0.010	0.005	-0.005	0.820*	1.098
	-0.09,0.03	-0.08,0.06	-0.01,0.02	-0.02,0.01	0.66,1.03	0.84,1.43
40+	-0.033	0.152***	-0.005	-0.014	0.822	1.282
	-0.11,0.05	0.04,0.27	-0.02,0.01	-0.04,0.01	0.61,1.12	0.80,2.05
Age at Interview	-0.036	0.078***	-0.002	-0.004	0.789**	0.932
	-0.09,0.02	0.02,0.13	-0.01,0.01	-0.02,0.01	0.63,0.98	0.74,1.17
Education (Ref: Low)						
Medium	-0.055	-0.058*	0.011	0.005	0.867	0.918
	-0.12,0.01	-0.12,0.00	-0.00,0.02	-0.01,0.02	0.67,1.11	0.72,1.16
High	-0.108**	-0.054	0.012	-0.006	0.901	0.908
	-0.20,-0.02	-0.14,0.03	-0.01,0.03	-0.02,0.01	0.65,1.26	0.65,1.27
Number of Partnerships (Ref: 0)						
1	-1.870***	-0.353*	-0.172	-0.025	0.962	0.266
	-3.03,-0.71	-0.74,0.03	-0.41,0.07	-0.11,0.06	0.81,1.14	0.04,1.75
2+	-1.883***	-0.338*	-0.169	-0.029	1.000	0.246
	-3.04,-0.72	-0.72,0.05	-0.41,0.07	-0.11,0.05		0.04,1.61
Ever Unemployed (1978-2001)	0.003	0.001	0.001	0.005	1.036	1.114
	-0.05,0.05	-0.05,0.05	-0.01,0.01	-0.01,0.02	0.86,1.24	0.91,1.37
Social Class at Birth - Manual	0.047	0.074**	0.005	0.002	1.105	1.504***
	-0.01,0.11	0.01,0.13	-0.01,0.02	-0.01,0.01	0.89,1.38	1.18,1.91
Ethnicity (White vs Non-White)	0.000	0.065	-0.018	0.004	1.012	1.357
	-0.15,0.14	-0.09,0.23	-0.05,0.01	-0.03,0.04	0.59,1.75	0.69,2.66
Special Educ. Treatment - Age 11	-0.011	-0.416***	-0.004	-0.033	1.022	0.657
	-0.23,0.21	-0.66,-0.17	-0.05,0.04	-0.08,0.01	0.44,2.39	0.26,1.64
Financial Hardship - Age 11	-0.096**	0.045	-0.001	-0.016*	0.698**	0.905
	-0.18,-0.01	-0.04,0.13	-0.02,0.02	-0.03,0.00	0.51,0.96	0.66,1.24
Overcrowding - Age 11	-0.010	0.012	-0.005	0.006	0.765*	0.960
	-0.09,0.07	-0.07,0.09	-0.02,0.01	-0.01,0.02	0.56,1.04	0.71,1.30

Housing Index (No access to…) - Age 11	-0.024	0.070*	-0.005	0.008	0.864	1.100
	-0.11,0.06	-0.01,0.15	-0.02,0.01	-0.01,0.02	0.63,1.18	0.82,1.48
Birth Weight	0.001	0.000	0.000	0.000	1.002	0.996
	-0.00,0.00	-0.00,0.00	-0.00,0.00	-0.00,0.00	1.00,1.01	0.99,1.00
Mother smoked during pregnancy	0.074***	0.054**	-0.007	-0.009	1.112	0.959
	0.02,0.12	0.00,0.10	-0.02,0.00	-0.02,0.00	0.92,1.34	0.79,1.17
BSGA Score - Age 11	0.003**	-0.002	0.000	0.000	1.003	0.988*
	0.00,0.01	-0.01,0.00	-0.00,0.00	-0.00,0.00	0.99,1.02	0.97,1.00
Parents interested in Edu - Age 11	0.019	-0.033	0.008	-0.001	0.986	0.812*
	-0.05,0.08	-0.10,0.03	-0.01,0.02	-0.01,0.01	0.77,1.26	0.64,1.03
Teenage Smoking - Age 16	0.066**	0.070**	-0.015**	-0.010*	1.219*	1.158
	0.01,0.12	0.02,0.12	-0.03,-0.00	-0.02,0.00	0.99,1.50	0.94,1.43
Rutter - Age 7	-0.010	0.004	-0.001	0.000	0.915*	1.070
	-0.04,0.02	-0.02,0.03	-0.01,0.00	-0.01,0.01	0.83,1.01	0.96,1.19
Rutter - Age 11	0.007	0.025*	-0.002	-0.002	1.057	0.970
	-0.02,0.03	-0.00,0.05	-0.01,0.00	-0.01,0.00	0.96,1.17	0.87,1.08
Parents' Years of Educ.	-0.022**	-0.009	-0.001	0.001	0.961	0.963
	-0.04,-0.00	-0.03,0.01	-0.00,0.00	-0.00,0.00	0.90,1.03	0.89,1.04
Mother stayed in school after min. age	-0.028	0.009	0.005	0.004	0.965	0.869
	-0.09,0.04	-0.06,0.08	-0.01,0.02	-0.01,0.02	0.76,1.23	0.66,1.15
Family Difficulties - Age 7	0.061	-0.022	0.021	0.012	1.498	1.046
	-0.12,0.24	-0.17,0.13	-0.02,0.06	-0.02,0.04	0.78,2.87	0.59,1.87
Parents Divorced by age 10/11	-0.123	-0.007	-0.027	-0.025*	0.971	0.908
	-0.30,0.05	-0.14,0.13	-0.07,0.01	-0.05,0.00	0.51,1.86	0.54,1.54
Times hospitalized - Age 11	-0.005	0.016	-0.001	-0.008**	0.901**	1.018
	-0.03,0.02	-0.01,0.04	-0.01,0.01	-0.01,-0.00	0.81,1.00	0.91,1.13
Out of school for 1+ months - Age 11	0.078	0.027	-0.009	0.011	0.779	1.299
	-0.03,0.19	-0.08,0.13	-0.03,0.01	-0.01,0.03	0.50,1.20	0.87,1.95
General Ability - Age 11	-0.003***	-0.002**	0.000	0.000	0.994	0.992**
	-0.01,-0.00	-0.00,-0.00	-0.00,0.00	-0.00,0.00	0.99,1.00	0.98,1.00
Enuresis - Age 7	-0.092**	0.037	0.002	0.025***	0.794	1.131
	-0.17,-0.01	-0.04,0.12	-0.02,0.02	0.01,0.04	0.59,1.07	0.84,1.53
Enuresis - Age 11	0.033	0.032	0.011	-0.028**	1.154	0.992
	-0.07,0.13	-0.09,0.15	-0.01,0.03	-0.05,-0.00	0.78,1.71	0.63,1.55
Physical Coordination Problems - Age 11	0.099***	0.147***	-0.011	0.008	1.169	1.747***
	0.03,0.17	0.07,0.22	-0.03,0.01	-0.01,0.02	0.88,1.55	1.31,2.33
Constant	13.097***	5.371***	1.064***	0.997***	70935.210**	110.955
	10.22,15.97	2.75,7.99	0.45,1.67	0.46,1.54	3.83,1.31e+09	0.00,4.29e+06
N	2,574	3,000	2,517	2,931	2,326	2,675

* p<0.10, ** p<0.05, *** p<0.01

Negative Controls

As 'negative controls' we use three variables from the 2002 biomedical survey: *hair color* (light brown and blond vs. dark brown and black), *ear tested first* (left vs. right), and *arm blood taken from* (left vs. right). These variables are then used as outcomes in the regression models instead of the biomarkers (using the same fertility variables, control variables, and multiple imputation techniques). These variables do not have a plausible mechanism of action that links them with fertility outcomes, other than confounding and/or measurement error. Table A8 reports descriptive statistics of the negative controls, and Figure A7, Figure A8, and Figure A9 report the regression results for number of children, age at first birth, and age at 'last' birth respectively. From these graphs, it is possible to observe that there is no association between fertility histories and negative controls as expected. Exceptions are associations for women who have 4 or more children, who are less likely to have their blood taken from their left arm, men who have 3 children (and a similar "marginally non-significant" pattern for men with 4 or more children), who are more likely to have their left ear tested first, suggesting that the relationship between parity and biomarkers is due to confounding and/or measurement error,

Table A8. Negative Controls

	**Men**		**Women**	
	**Mean or %**	**N**	**Mean or %**	**N**
Dark Hair Color (Ref: Light)	53.3	4,227	42.4	4,410
Left Ear Tested First (Ref: Rigt)	89.0	4,602	90.3	4,658
Blood Taken from Left Arm (Ref: Right)	50.1	3,856	49.7	3,864
N in Biomedical Survey	*4,665*		*4,712*	

Figure A7. Number of Children and Negative Controls

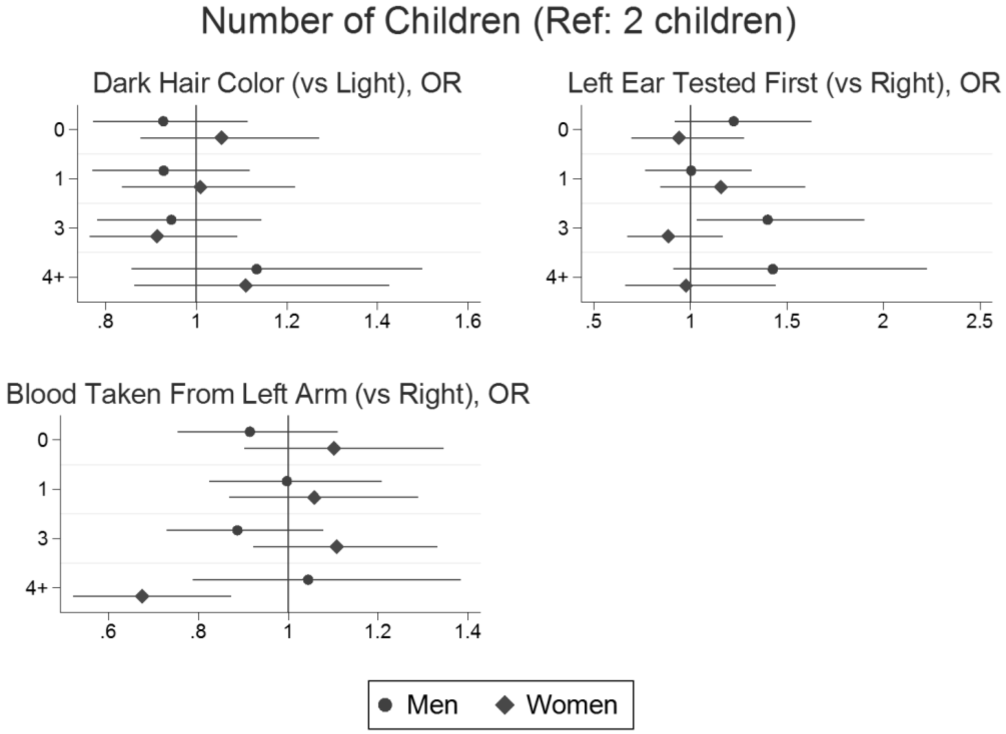

Figure A8. Age at First Birth and Negative Controls

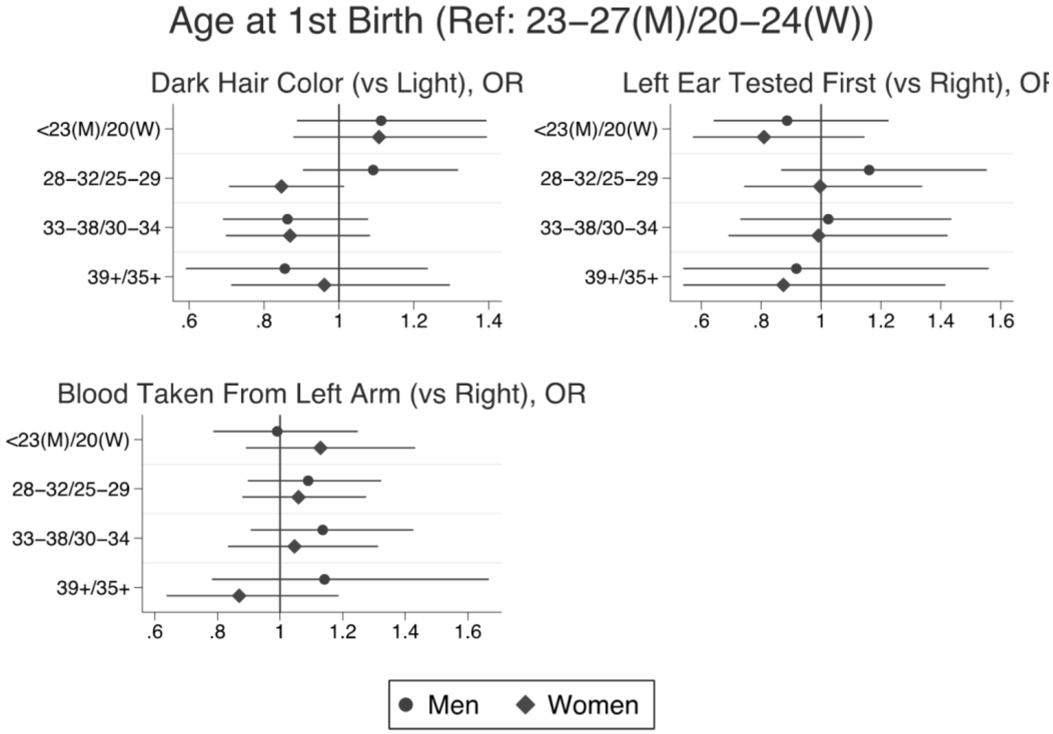

Figure A9. Age at Last Birth and Negative Controls

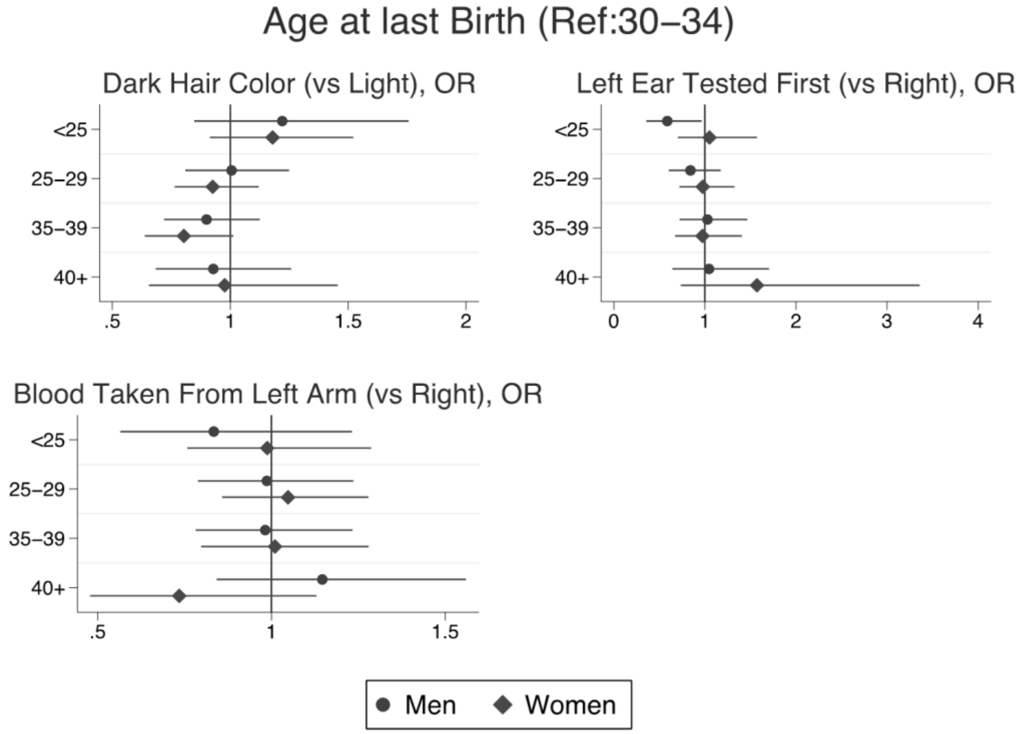

Complete Case Analysis

Figure A10. Number of Children and Biomarkers (Complete Case Sample)

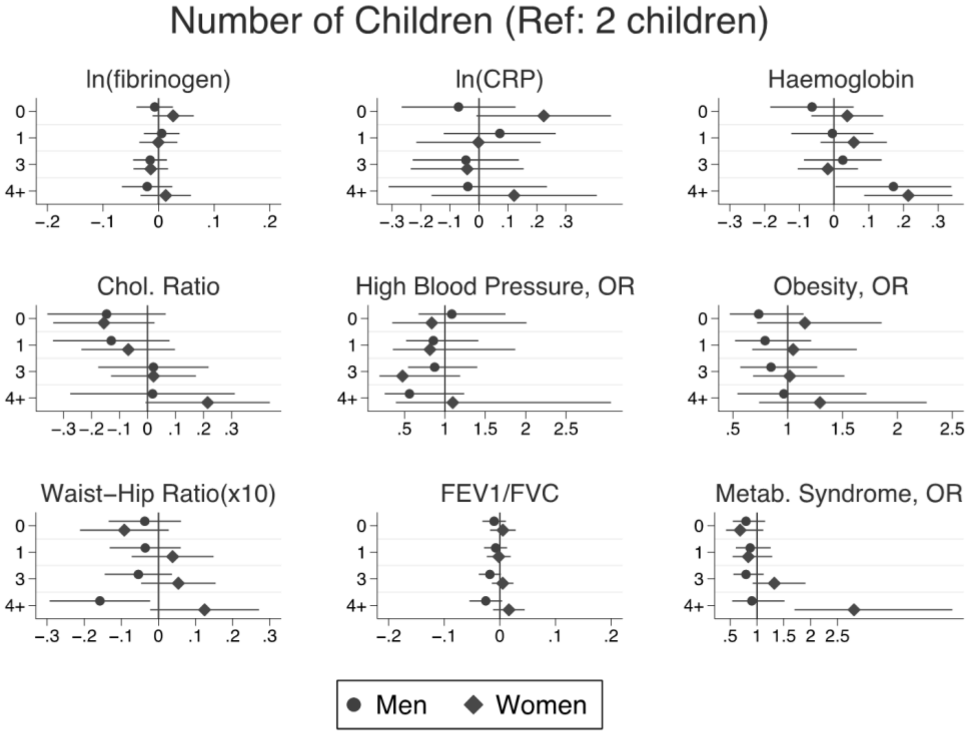

Figure A11. Age at First Birth and Biomarkers (Complete Case Sample)

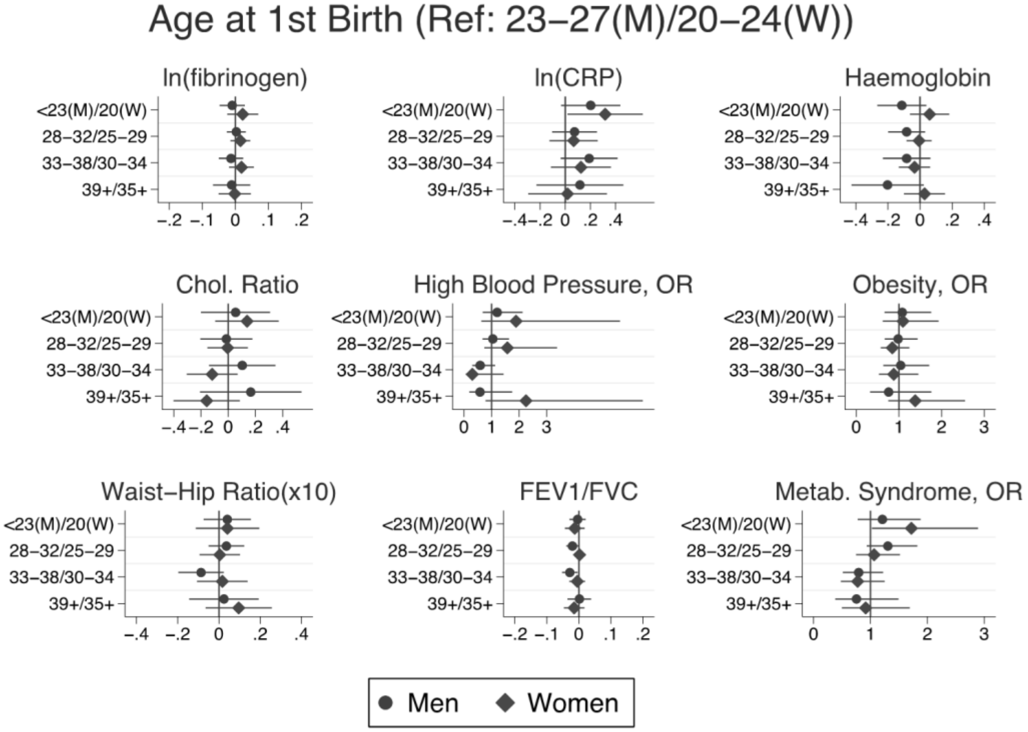

Figure A12. Age at Last Birth and Biomarkers (Complete Case Sample)

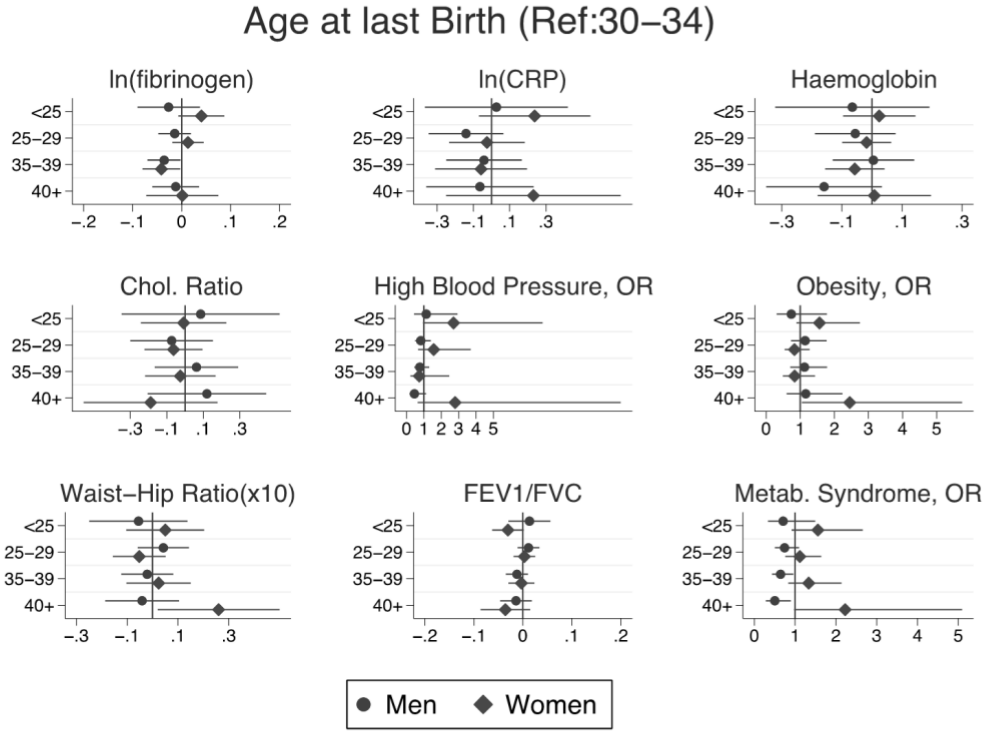

Comparison with Self-reported Health

Figure A13. Number of Children and Biomarkers + Self-Reported Health

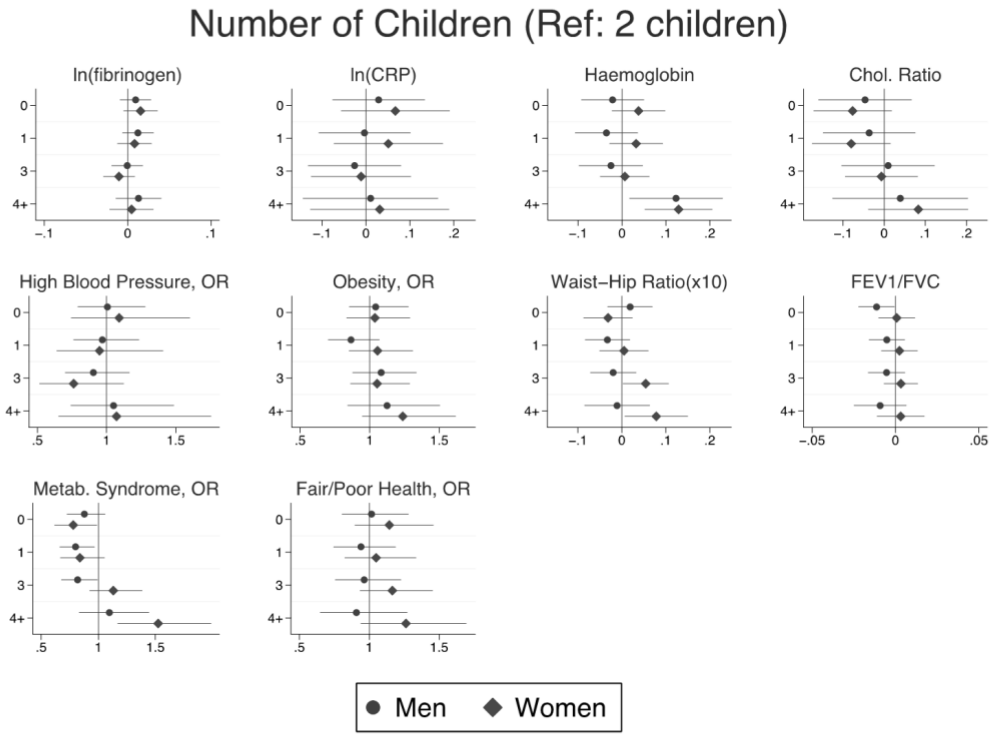

Figure A14. Age at First Birth and Biomarkers + Self-Reported Health

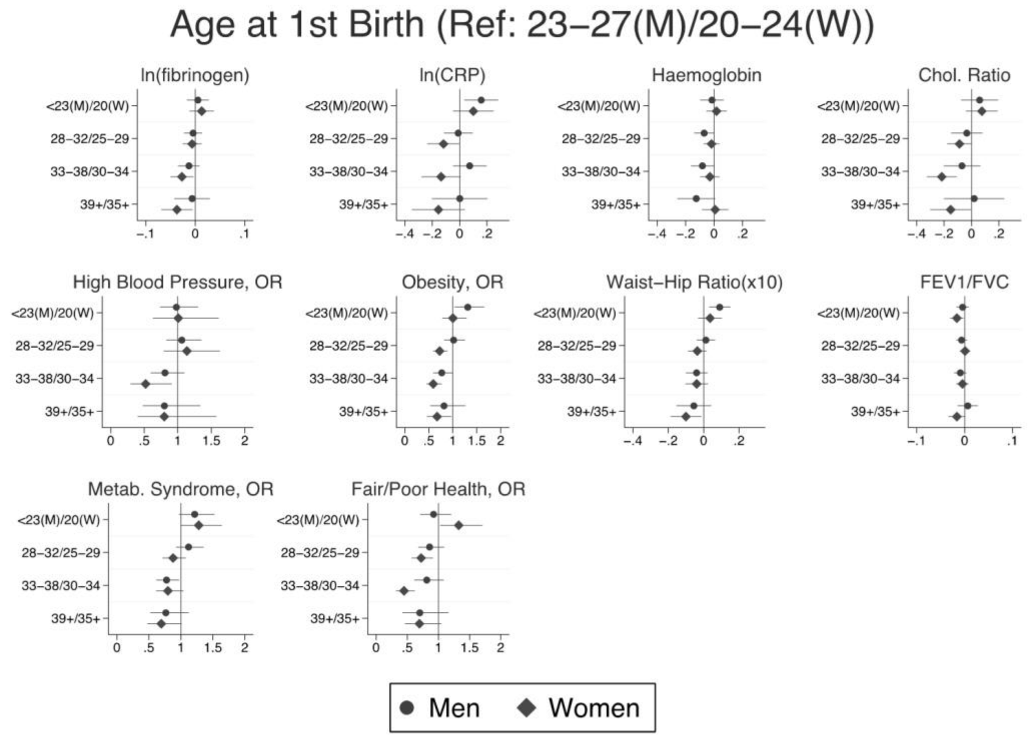

Figure A15. Age at Last and Biomarkers + Self-Reported Health

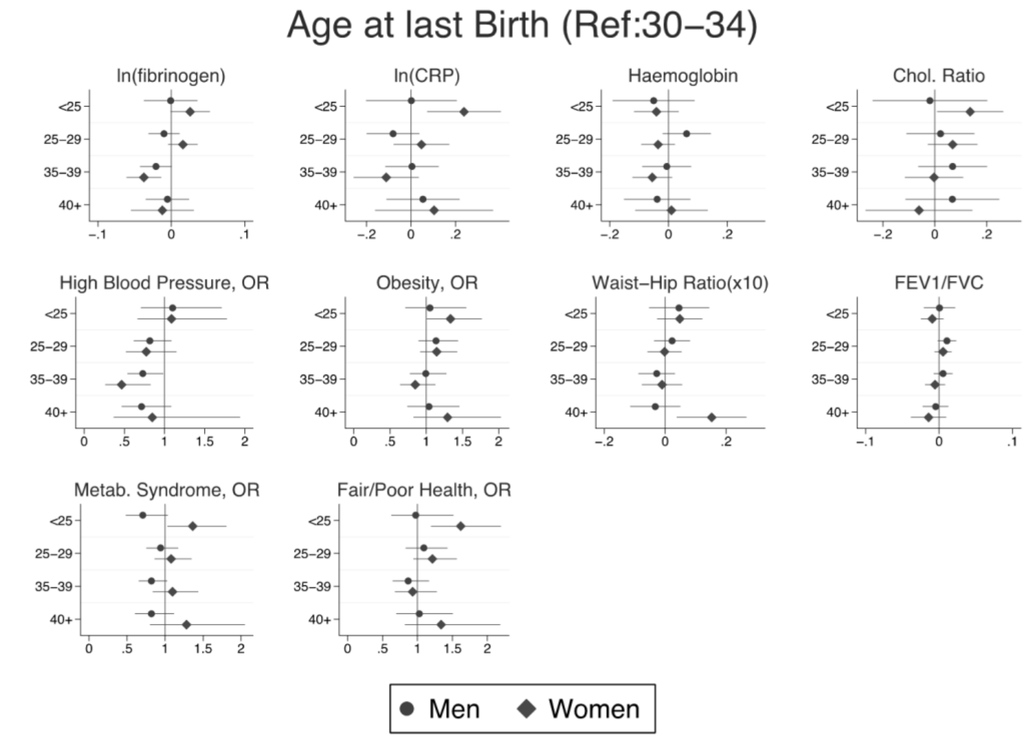

Multiple Testing – Bonferroni Correction

Table A9. Biomarkers and Number of Children

	**log(Fibrinogen)**		**log(C-Reactive Protein)**		**Glycated Haemoglobin**		**Cholesterol Ratio (Tot./HDL)**	
	**Men**	**Women**	**Men**	**Women**	**Men**	**Women**	**Men**	**Women**
**Number of Children, Ref: 2 Children**	*B/CI*	*B/CI*	*B/CI*	*B/CI*	*B/CI*	*B/CI*	*B/CI*	*B/CI*
0	0.009	0.016	0.029	0.067	-0.021	0.038	-0.046	-0.077
	-0.01,0.03	-0.00,0.04	-0.08,0.13	-0.06,0.19	-0.09,0.05	-0.02,0.10	-0.16,0.07	-0.17,0.02
1	0.012	0.008	-0.003	0.051	-0.035	0.032	-0.036	-0.080*
	-0.01,0.03	-0.01,0.03	-0.11,0.10	-0.07,0.17	-0.11,0.04	-0.03,0.09	-0.15,0.08	-0.18,0.02
3	-0.001	-0.01	-0.026	-0.011	-0.025	0.007	0.009	-0.007
	-0.02,0.02	-0.03,0.01	-0.13,0.08	-0.12,0.10	-0.10,0.05	-0.05,0.06	-0.10,0.12	-0.09,0.08
4+	0.013	0.005	0.01	0.031	0.123**	**0.129******	0.039	0.083
	-0.01,0.04	-0.02,0.03	-0.14,0.16	-0.13,0.19	0.02,0.23	0.05,0.21	-0.13,0.20	-0.04,0.20
N	3,699	3,713	3,709	3,712	3,824	3,820	3,770	3,766

	**High Blood Pressure**		**Obesity**		**Waist-Hip Ratio*10**		**FEV1/FVC**		**Metabolic Syndrome**	
	**Men**	**Women**	**Men**	**Women**	**Men**	**Women**	**Men**	**Women**	**Men**	**Women**
**Number of Children, Ref: 2 Children**	*OR/CI*	*OR/CI*	*OR/CI*	*OR/CI*	*B/CI*	*B/CI*	*B/CI*	*B/CI*	*OR/CI*	*OR/CI*
0	1.006	1.092	1.044	1.037	0.019	-0.031	-0.011**	0.001	0.877	0.780**
	0.79,1.28	0.74,1.60	0.85,1.28	0.83,1.29	-0.03,0.07	-0.09,0.02	-0.02,-0.00	-0.01,0.01	0.73,1.06	0.62,0.99
1	0.97	0.95	0.866	1.056	-0.033	0.005	-0.005	0.002	0.800**	0.839
	0.76,1.23	0.64,1.41	0.70,1.07	0.85,1.31	-0.08,0.02	-0.05,0.06	-0.02,0.01	-0.01,0.01	0.66,0.97	0.67,1.05
3	0.904	0.762	1.082	1.053	-0.019	0.054**	-0.005	0.003	0.819**	1.13
	0.70,1.16	0.52,1.12	0.88,1.34	0.86,1.29	-0.07,0.03	0.00,0.11	-0.02,0.01	-0.01,0.01	0.68,0.99	0.92,1.38
4+	1.049	1.072	1.125	1.237	-0.011	0.078**	-0.009	0.003	1.096	**1.524******
	0.74,1.49	0.65,1.76	0.84,1.50	0.95,1.62	-0.08,0.06	0.01,0.15	-0.02,0.01	-0.01,0.02	0.83,1.44	1.17,1.99
N	4,424	4,470	4,405	4,473	4,444	4,517	4,343	4,421	3,980	4,011

Note: * p<0.1, ** p<0.05, *** p<0.01, ****** p<0.0055**

Table A10. Biomarkers and Age at 1st Birth

	log(Fibrinogen)		log(C-Reactive Protein)		Glycated Haemoglobin		Cholesterol Ratio (Tot./HDL)	
	**Men**	**Women**	**Men**	**Women**	**Men**	**Women**	**Men**	**Women**
**Age at 1st Birth, Ref: 23-27 (M)/20-24 (W)**	*B/CI*	*B/CI*	*B/CI*	*B/CI*	*B/CI*	*B/CI*	*B/CI*	*B/CI*
<23(M)-<20(W)	0.005	0.013	0.157**	0.100	-0.015	0.018	0.059	0.075
	-0.02,0.03	-0.01,0.04	0.03,0.28	-0.05,0.25	-0.10,0.07	-0.05,0.09	-0.07,0.19	-0.04,0.19
28-32 (M) / 25-29 (W)	-0.004	-0.006	-0.01	-0.118**	-0.069**	-0.017	-0.034	-0.088*
	-0.02,0.01	-0.03,0.01	-0.11,0.09	-0.23,-0.00	-0.14,-0.00	-0.07,0.04	-0.15,0.08	-0.18,0.00
33-38 (M) / 30-34 (W)	-0.013	-0.027**	0.074	-0.135*	-0.081**	-0.03	-0.069	**-0.217****
	-0.03,0.01	-0.05,-0.00	-0.05,0.20	-0.28,0.01	-0.16,-0.00	-0.10,0.04	-0.20,0.06	-0.33,-0.11
39+ (M) / 35+ (W)	-0.007	-0.037**	0.001	-0.155	-0.125*	0.008	0.019	-0.152**
	-0.04,0.03	-0.07,-0.01	-0.20,0.20	-0.35,0.04	-0.26,0.01	-0.08,0.10	-0.20,0.24	-0.30,-0.00
N	2,823	3,035	2,833	3,034	2,908	3,118	2,873	3,072

	High Blood Pressure		Obesity		Waist-Hip Ratio*10		FEV1/FVC		Metabolic Syndrome	
	**Men**	**Women**	**Men**	**Women**	**Men**	**Women**	**Men**	**Women**	**Men**	**Women**
**Age at 1st Birth, Ref: 23-27 (M)/20-24 (W)**	*OR/CI*	*OR/CI*	*OR/CI*	*OR/CI*	*B/CI*	*B/CI*	*B/CI*	*B/CI*	*OR/CI*	*OR/CI*
<23(M)-<20(W)	0.978	1.009	1.310**	1.003	**0.091****	0.036	-0.004	-0.016**	1.216*	1.279*
	0.74,1.30	0.63,1.61	1.04,1.65	0.79,1.28	0.03,0.15	-0.03,0.10	-0.02,0.01	-0.03,-0.00	0.97,1.52	1.00,1.64
28-32 (M) / 25-29 (W)	1.06	1.136	1.017	**0.725****	0.013	-0.036	-0.006	0.001	1.119	0.879
	0.83,1.35	0.79,1.63	0.83,1.25	0.59,0.89	-0.04,0.06	-0.09,0.02	-0.02,0.00	-0.01,0.01	0.93,1.35	0.71,1.08
33-38 (M) / 30-34 (W)	0.81	0.520**	0.770**	**0.592****	-0.04	-0.039	-0.009	-0.005	0.776**	0.798*
	0.60,1.10	0.30,0.91	0.59,1.00	0.45,0.77	-0.10,0.02	-0.10,0.02	-0.02,0.00	-0.02,0.01	0.62,0.97	0.61,1.04
39+ (M) / 35+ (W)	0.801	0.798	0.818	0.671**	-0.055	-0.100**	0.006	-0.016*	0.766	0.694*
	0.48,1.33	0.40,1.57	0.53,1.26	0.47,0.97	-0.15,0.04	-0.19,-0.01	-0.01,0.03	-0.03,0.00	0.53,1.12	0.48,1.01
N	3,310	3,632	3,299	3,629	3,325	3,665	3,255	3,588	3,007	3,260

Note: * p<0.1, ** p<0.05, *** p<0.01, ****** p<0.0055**

Table A11. Biomarkers and Age at last Birth

	log(Fibrinogen)		log(C-Reactive Protein)		Glycated Haemoglobin		Cholesterol Ratio (Tot./HDL)	
	**Men**	**Women**	**Men**	**Women**	**Men**	**Women**	**Men**	**Women**
**Age at Last Birth, Ref: 30-34**	*B/CI*	*B/CI*	*B/CI*	*B/CI*	*B/CI*	*B/CI*	*B/CI*	*B/CI*
<25	-0.001	0.026*	0.002	**0.239********	-0.051	-0.041	-0.019	0.136**
	-0.04,0.04	-0.00,0.05	-0.20,0.21	0.07,0.40	-0.19,0.09	-0.12,0.03	-0.24,0.20	0.01,0.26
25-29	-0.01	0.016	-0.081	0.047	0.062	-0.035	0.022	0.069
	-0.03,0.01	-0.00,0.04	-0.20,0.04	-0.08,0.17	-0.02,0.14	-0.09,0.02	-0.11,0.15	-0.03,0.16
35-39	-0.021*	**-0.037********	0.004	-0.112	-0.006	-0.055	0.069	-0.003
	-0.04,0.00	-0.06,-0.01	-0.12,0.12	-0.26,0.03	-0.09,0.08	-0.12,0.01	-0.06,0.20	-0.12,0.11
40+	-0.005	-0.012	0.054	0.104	-0.038	0.01	0.067	-0.061
	-0.03,0.02	-0.05,0.03	-0.11,0.22	-0.16,0.37	-0.15,0.07	-0.11,0.13	-0.11,0.25	-0.27,0.14
N	2,197	2,501	2,205	2,500	2,259	2,565	2,240	2,530

	High Blood Pressure		Obesity		Waist-Hip Ratio*10		FEV1/FVC		Metabolic Syndrome	
	**Men**	**Women**	**Men**	**Women**	**Men**	**Women**	**Men**	**Women**	**Men**	**Women**
**Age at Last Birth, Ref: 30-34**	*OR/CI*	*OR/CI*	*OR/CI*	*OR/CI*	*B/CI*	*B/CI*	*B/CI*	*B/CI*	*OR/CI*	*OR/CI*
<25	1.101	1.087	1.051	1.334**	0.045	0.048	0.001	-0.009	0.708*	1.365**
	0.71,1.71	0.67,1.78	0.71,1.55	1.01,1.77	-0.05,0.14	-0.03,0.12	-0.02,0.02	-0.02,0.01	0.48,1.03	1.03,1.81
25-29	0.819	0.773	1.134	1.145	0.022	-0.002	0.011*	0.005	0.941	1.079
	0.62,1.09	0.52,1.15	0.90,1.44	0.92,1.43	-0.04,0.08	-0.06,0.05	-0.00,0.02	-0.01,0.02	0.76,1.17	0.86,1.35
35-39	0.729**	0.467***	0.995	0.849	-0.028	-0.01	0.005	-0.005	0.820*	1.098
	0.54,0.98	0.26,0.83	0.78,1.28	0.64,1.12	-0.09,0.03	-0.08,0.06	-0.01,0.02	-0.02,0.01	0.66,1.03	0.84,1.43
40+	0.713	0.847	1.038	1.295	-0.033	0.152***	-0.005	-0.014	0.822	1.282
	0.47,1.08	0.37,1.94	0.74,1.46	0.83,2.03	-0.11,0.05	0.04,0.27	-0.02,0.01	-0.04,0.01	0.61,1.12	0.80,2.05
N	2,555	2,960	2,556	2,973	2,574	3,000	2,517	2,931	2,326	2,675

Note: * p<0.1, ** p<0.05, *** p<0.01, ****** p<0.0055**
